# Environmental Factors Exacerbate Parkinsonian Phenotypes in an Asian-Specific Knock-In LRRK2 Risk Variant in Mice

**DOI:** 10.3390/ijms26083556

**Published:** 2025-04-10

**Authors:** Zoë Bichler, Sarivin Vanan, Zhiwei Zhang, Qianying (Sally) Dong, Jolene Wei Ling Lee, Chengwu Zhang, Liting Hang, Mei Jiang, Parasuraman Padmanabhan, Wuan Ting Saw, Zhidong Zhou, Balázs Gulyás, Kah Leong Lim, Li Zeng, Eng King Tan

**Affiliations:** 1 Behavioural Neuroscience Lab, Research Department, National Neuroscience Institute, Singapore 308433, Singapore; zoe.bichler@jax.org (Z.B.); sallydong1229@gmail.com (Q.D.); 2 Center for Biometric Analysis, The Jackson Laboratory, Bar Harbor, ME 04609, USA; 3 Neural Stem Cell Research Lab, Research Department, National Neuroscience Institute, Singapore 308433, Singapore; sarivinv001@e.ntu.edu.sg (S.V.); zhi_wei_zhang@nni.com.sg (Z.Z.); jolene_wl_lee@u.duke.nus.edu (J.W.L.L.); jiangm526@outlook.com (M.J.); 4 Lee Kong Chian School of Medicine, Nanyang Technological University, Singapore 308232, Singapore; ppadmanabhan@ntu.edu.sg (P.P.); balazs.gulyas@ntu.edu.sg (B.G.); kahleong.lim@ntu.edu.sg (K.L.L.); 5 Neurodegeneration Research Lab, Research Department, National Neuroscience Institute, Singapore 308433, Singapore; chengwu_zhang@sxmu.edu.cn (C.Z.); litinghang@gmail.com (L.H.); 6 Neuroscience and Behavioral Disorders Program, DUKE-NUS Graduate Medical School, Singapore 169857, Singapore; wuan_ting_saw@nni.com.sg (W.T.S.); zhidong_zhou@nni.com.sg (Z.Z.); 7 Dongguan Key Laboratory of Stem Cell and Regenerative Tissue Engineering, Department of Human Anatomy, Dongguan Campus, Guangdong Medical University, Dongguan 523808, China; 8 Translational Therapeutics Lab, Research Department, National Neuroscience Institute, Singapore 169856, Singapore; 9 Research Department, National Neuroscience Institute, Singapore General Hospital (SGH) Campus, Singapore 168581, Singapore; 10 Department of Neurology, National Neuroscience Institute, Singapore 308433, Singapore

**Keywords:** Parkinson’s disease, LRRK2, Asian specific variant, environmental toxins, physiological stress, oxidative stress, behaviour

## Abstract

Parkinson’s disease (PD) is a neurodegenerative disorder affecting nearly 10 million people worldwide, and for which no cure is currently known. Mutations in the Leucine-Rich Repeat Kinase 2 (LRRK2) gene, age, as well as environmental factors such as neurotoxin exposure and stress, are known to increase the risk of developing the disease in humans. To investigate the role of a specific Asian variant of the LRRK2 gene to induce susceptibility to stress and trigger PD phenotypes with time, knock-in (KI) mice bearing the human LRRK2 R1628P risk variant have been generated and studied from 2 to 16 months of age in the presence (or absence) of stress insults, including neurotoxin injections and chronic mild stress applied at 3 months of age. Pathophysiological and behavioural phenotypes have been measured at different ages and primary neurons and fibroblast cells were cultured from the KI mouse line and treated with H_2_O_2_ to study susceptibility towards oxidative stress *in vitro*. KI mice displayed specific PD features and these phenotypes were aggravated by environmental stresses. In particular, KI mice developed locomotion impairment and increased constipation. In addition, dopamine-related proteins were dysregulated in KI mice brains: Dopamine transporter (DAT) was decreased in the midbrain and striatum and dopamine levels were increased. Primary fibroblast cells and cortical neurons from KI mice also displayed increased susceptibility to oxidative stress. Therefore, the LRRK2 R1628P KI mice are an excellent model to study the progressive development of PD.

## 1. Introduction

Parkinson’s disease (PD) is globally the second most common neurodegenerative disorder [1]. It is diagnosed by classical motor symptoms such as bradykinesia, myotonia, resting tremors, abnormal gait, and postural instability [2,3]. At this stage of the disease, already half of dopaminergic neurons are depleted in the substantia nigra (SN), the total amount of dopamine (DA) decreases by more than 70% in the brain of patients, and Lewy body aggregates are present in the substantia nigra-striatum system [4]. In addition, several nonmotor symptoms have usually preceded the motor symptoms by years, including olfactory deficits, mood disorders, sleep disturbances, difficulty swallowing and cognitive impairment [5,6,7]. Unfortunately, at the time of diagnosis, palliative drugs that slow down the irreversible course of the disease are the only way to treat the disease, as no curative therapies exist yet.

Clinically, PD can be subdivided into familial and sporadic PD. While familial PD is caused by genetic mutations [8], the aetiology of sporadic PD remains elusive and epigenetic factors may have a significant impact on the development of the disease. Importantly, the age at onset, severity of symptoms, and rate of progression of PD vary dramatically among patients, increasing the complexity of the clinical prognosis [9].

Several aetiological factors correlating to the development of PD have already been identified [10]. Like with ageing, the risk of developing PD increases with early exposure to neurotoxins like pesticides and herbicides, as well as viruses and bacteria. Psychological stresses such as chronic stress have also been shown to play a role in molecular processes of neurodegeneration and cognition [11], and studies have emphasized their role in triggering or exacerbating PD symptoms in humans [12,13] and mice [14].

In about 10% of PD cases, genetic variants have been directly correlated with the disease. As of today, over 20 known pathogenic genes are related to PD including Parkin, Alpha Synuclein (SNCA), PTEN Induced Kinase 1 (PINK1) and Leucine-Rich Repeat Kinase 2 (LRRK2) [8,15,16]. Thus far, only LRRK2 is associated with both familial PD and idiopathic PD [15,17] and accounts for approximately 1% of all PD cases [18,19]. LRRK2 is highly expressed in brain regions such as the hippocampus, cortex, amygdala and striatum, where it is generally associated with the regulation of protein translation, axonal growth and ageing in the nervous system [20,21].

In humans, at least 20 different LRRK2 variants have been described as risk factors for PD, of which the gain of function mutation G2019S is the most penetrant. Among the LRRK2 variants, LRRK2 R1628P is a risk variant located in the COR domain [22]. It is a substitution whereby the basic and polar arginine is replaced with a neutral and non-polar proline [23]. While the pathologic mechanisms behind the LRRK2 R1628P risk variant remain unclear, some evidence indicates that it may indirectly increase LRRK2 kinase activity leading eventually to neuronal death [22]. It is relatively prevalent in East Asian populations and is found in approximately 5 to 11% of PD cases in countries such as China, Singapore, Taiwan and Thailand [24]. Of significant interest, it has also been shown to increase the likelihood of developing PD by two-fold [25]. However, apart from one known causative gene (SNCA) [26], evidence from epidemiological studies shows that all genetic variants do not cause PD on their own but rather increase the risk of developing it. As a consequence, genetic mutations including LRRK2 variants may well set a basis upon which other stress-related mechanisms are critical key triggers of PD [27].

A large number of animal models have been used in an attempt to understand LRRK2-linked Parkinsonism and mimic the disorder [28,29] by either overexpressing [30,31,32] or knocking out specific LRRK2 genes [33,34,35]. Most of these models help shed light on some mechanisms but fail to recapitulate the behavioural and neuroanatomical features of PD fully [28,29]. On the other hand, neurotoxic models help understand molecular dysfunctions, but they do not translate the progressive development of human PD, dismissing important mechanisms of the prodromal aspect of the disease.

In this study, using a novel LRRK2 KI mouse model for the R1628P variant, we tried to consider the appearance of the symptoms with time by combining a genetic approach with environmental insults and ageing. We found that mice carrying the R1628P risk variant are more prone to develop clinical features of PD, in particular when the mice were exposed as young adults to neurotoxic and psychologically chronic mild stresses.

## 2. Results

### 2.1. R1628P Risk Variant in Mice Differentially Regulates Key PD-Related Proteins

**Figure 1:**
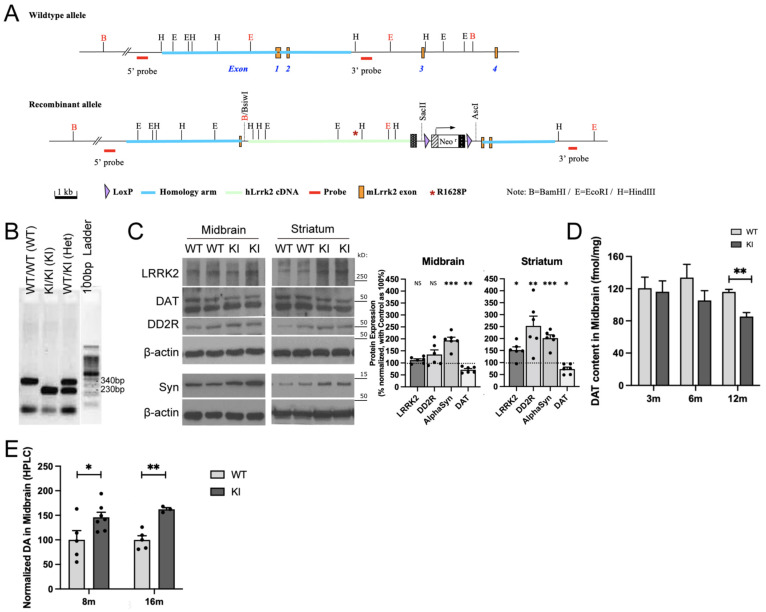
R1628P risk variant in mice differentially regulates key PD-related proteins. (**A**) Schematic representation of the WT and KI transgenic constructs. (**B**) PCR genotyping analysis of LRRK2 R1628P mouse model. Lane 1: WT/WT (WT), Lane 2: KI/KI (KI), Lane 3: WT/KI (Het), Lane 4: DNA ladder. (**C**) Western blot analysis of LRRK2, DAT (upper band), DD2R and α-synuclein in the midbrain and striatum of 8-month-old WT and KI mice. Protein levels normalized to aged-matched WT controls (n = 6/group). (**D**) Autoradiograph evaluation of DAT in the midbrain of KI mice and WT controls at age 3, 6 and 12 months old (n = 3/group). (**E**) HPLC analysis of midbrain DA levels in KI mice at 8 and 16 months old. Levels normalized to aged-matched WT controls (n = 3 to 4/group). Data are means ± SEM; One-way ANOVA followed by Bonferroni post hoc comparisons, * *p* < 0.05, **p < 0.01, ***p < 0.001.

Human R1628P cDNA was generated and inserted into the endogenous mouse *Lrrk2* exon (Figure 1A, Supplementary Document S1). Knock-In (KI) mice expressed the human LRRK2 R1628P risk variant protein while the wildtype non-transgenic mice (WT) expressed the endogenous mouse LRRK2 protein, with both respective human and mouse genes working under the same endogenous promoter. Genotyping was performed on tail samples and Polymerase chain reaction (PCR) amplification was carried out to confirm the transgenicity (Figure 1B). The transgene segregated well and the mouse line bred normally. WT and KI mice were indistinguishable in terms of body weight and dysmorphology was not observed in major organs.

Western blot, autoradiography and high-performance liquid chromatography (HPLC) were used to analyse the amount of LRRK2, dopamine D2 receptor (DD2R), α-synuclein, and dopamine transporter (DAT) proteins in specific brain regions of LRRK2 R1628P KI and WT mice at different ages.

Since LRRK2 R1628P is a PD risk variant (and not a causal gene like SNCA) it only predisposes KI mice to PD instead of outright inducing it [26]. Therefore, mice were first aged to 8 months old to allow time for biochemical PD phenotypes to begin. Subsequently, Western blot was performed on midbrain and striatal tissue from WT and KI mice. Total LRRK2 levels quantified by Western Blot with an antibody recognizing the C-terminal of both the mouse and human protein were increased in the striatum of 8-month-old KI mice (Figure 1C); LRRK2 protein level was however not significantly changed in the midbrain.

DAT was reduced in the midbrain and striatum in LRRK2 R1628P KI mice aged 8 months (Figure 1C), and the fine quantification of DAT by autoradiography confirmed the significant decrease in KI mice at 12 months old (Figure 1D). In addition, KI mice displayed increased expression of α-synuclein in both midbrain and striatum and elevated DD2R in the striatum (Figure 1C). Dopamine (DA) quantified by HPLC was increased in midbrain tissues of the KI mice as early as 8 months old and continued to remain increased at 16 months old (Figure 1E). Overall, adult LRRK2 R1628P KI mice displayed several of the specific pathophysiological features associated with PD.

### 2.2. Combination of Paraquat/Maneb and Unpredictable Chronic Mild Stress Accelerates Behavioural Deficits in LRRK2 R1628P KI Mice

Environmental factors may play a significant role in the progression of PD. A treatment combining the agricultural neurotoxins paraquat (a herbicide) and maneb (a fungicide) has indeed been previously shown to accelerate PD phenotypes, by inducing dopaminergic neuronal loss and subsequent locomotor deficits in mice [36,37]. In addition, studies performed on A53T transgenic mice showed that unpredictable chronic mild stress (UCMS) reduced dopaminergic neurons and led to motor disabilities [38].

**Figure 2:**
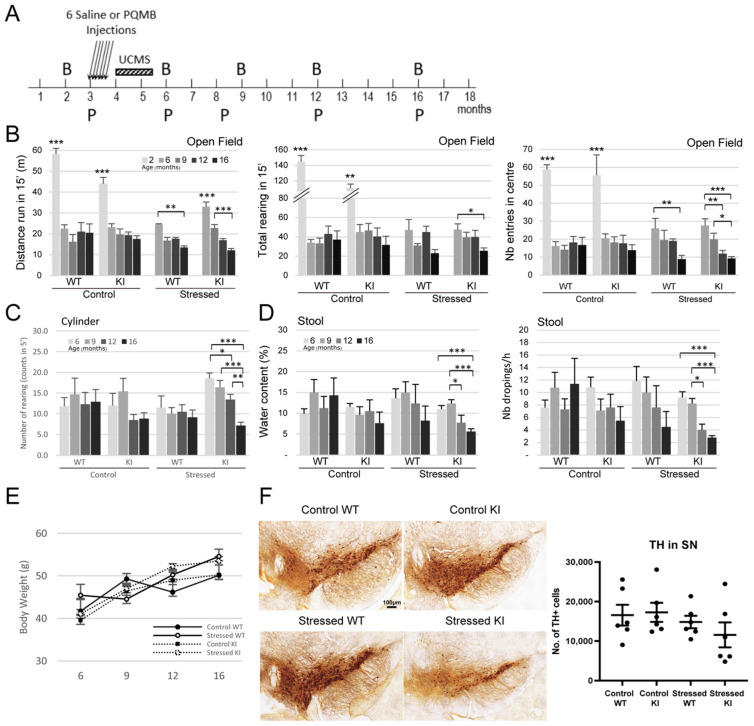
Combination of Paraquat/Maneb and unpredictable chronic mild stress accelerates behavioural deficits in LRRK2 R1628P KI mice (**A**) Experimental outline. PQMB: Paraquat/Maneb, UCMS: Unpredictable chronic mild stress, B: Behavior analysis, P: Protein analysis. Six PQMB or saline injections were given at 3 months old and the PQMB-treated group underwent additional six weeks of UCMS. (**B**) Open field test at 2, 6, 9, 12, and 16 months old; left: distance travelled, middle: total number of rearing, right: number of entries in the centre. Two-month-old mice outperform 6-, 9-, 12-, and 16-month-old mice in both WT and KI groups (n number: Control WT-9/Control KI-8/Stressed WT-8/Stressed KI-16) [Tests of between-subject effects: Distance genotype + age combination effect, F(4,18) = 2.714; *p* < 0.033/Distance age effect, F(4,18) = 13.264; *p* < 0.01/Rearing age effect, F(4,18) = 6.631; *p* < 0.01/Center entries age effect, F(4,18) = 10.663; *p* < 0.01]. (**C**) Cylinder test at 6, 9, 12, and 16 months old (n number: Control WT-8/Control KI-9/Stressed WT-6/Stressed KI-23) [Tests of between-subject effects: Rearing genotype + stress combination effect, F(1,16) = 5.132; *p* < 0.025]. (**D**) Analysis of stool water content and number of stool droppings at 6, 9, 12, and 16 months old (n number: Control WT-8/Control KI-8/Stressed WT-6/Stressed KI-15) [Tests of between-subject effects: Water content genotype effect, F(1,15) = 11.048; *p* < 0.001/Water content age effect, F(1,15) = 2.591; *p* < 0.056/Nb of droppings genotype effect, F(1,15) = 5.626; *p* < 0.019/Nb of droppings age effect, F(1,15) = 4.362; *p* < 0.006]. (**E**) Body weight data over time. (**F**) Number of TH+ cells in substantia nigra using DAB staining (n = 6/group), scale bar: 100 µm. Data are means ± SEM, Two-way ANOVA followed by Bonferroni post hoc comparisons, and repeated measures considered for (**B**–**E**); * *p* < 0.05, ** *p* < 0.01, *** *p* < 0.001.

In this longitudinal study, both chemical and psychological insults have been combined to better mimic the human condition. Six paraquat/maneb injections at relatively low doses were administered intraperitoneally to 3-month-old LRRK2 R1628P WT and KI mice, followed by 6 weeks of UCMS. The overall paraquat/maneb and UCMS treatment is here considered as the “stress treatment”. Behavioural phenotyping and biochemical sampling were performed at regular intervals from 2 to 16 months old (Figure 2A).

WT and KI mice were tested at two months old in the open field before receiving any treatment; all mice were very active as expected by their age (Figure 2B). Groups of mice were then tested at 6, 9, 12 and 16 months old. The activity of Control-WT and Control-KI mice did not change dramatically with aging from 6 months old onwards: there was no difference in the distance run, number of rearing and number of entries into the centre over the 10-month period (Figure 2B). However, stress affected significantly motor abilities. Interestingly, Stressed-KI mice seemed to travel a greater distance than Stressed-WT mice at 6 months old, suggesting hyperactivity. Stressed-WT mice travelled significantly less at 16 months old than they did at 6 months old, and this deficit was even more pronounced in Stressed-KI mice which displayed a significant reduction in distance travelled at just 9 months old, and a further decrease at 16 months old. While no decrease in rearing was observed in the WT mice, the KI mice showed significantly less rearing at 16 months old. Both WT and KI mice in the stressed group exhibited a significantly smaller number of entries into the centre of the arena, with KI mice showing this deficit 4 months earlier than the WT (12 months versus 16 months old) (Figure 2B). In the Cylinder Test, an assay measuring vertical movement in a small arena in 5 min, a considerable decrease was observed in the Stressed-KI mice beginning at 12 months old (Figure 2C), consistent with the Open Field Test data. Constipation is one of the prodromal symptoms of PD and can develop many years before clinical diagnosis [39]. Stool analysis was performed at different time points in this study, and the water content of collected faeces and the number of droppings were used as a proxy for gastrointestinal dysfunctions. Interestingly, the gastrointestinal system of Control-WT and KI mice seemed to be unaffected (Figure 2D). However, stress treatment affected the KI mice which display a condition close to constipation with decreases in both water content and number of droppings. Taken together, these data support the idea that individuals bearing a known genetic risk factor may be more susceptible to stress events and develop earlier and/or stronger a disorder linked to this risk.

In order to support the behavioural phenotyping data, mice were euthanized and brain samples were stained to investigate specific cellular biomarkers. In particular, the total number of dopaminergic neurons counted via the Tyrosine hydroxylase (TH) marker in the *substantia nigra pars compacta* (SNpc) was slightly decreased in 16-month-old Stressed-KI mice as compared to the other group of animals, although this was not significant (Figure 2F). It is possible that this mild decrease accounts for the locomotion impairment and constipation (a prodromal PD symptom) observed in the Stressed-KI mice; perhaps the decrease would be more crucial at a later age, as it may indicate an ongoing slow degenerating phenomenon. It is important to note that TH loss remains modest in other genetically modified mouse models for PD, even in LRRK2 mice bearing the G2019S mutation, which is a more penetrant LRRK2 variant [40,41].

### 2.3. Oxidative Stress Modulates RAB10, AKT, and ERK Activity in LRRK2 R1628P WT and KI Primary Fibroblast Cells and Primary Cortical Neurons

Oxidative stress has been implicated in the LRRK2 pathogenesis of PD. Previous studies show that LRRK2 overexpression in cortical neurons leads to the increased production of reactive oxygen species (ROS) which could accelerate dopaminergic neuronal death [42]. Interestingly, LRRK2 expression has also been shown to be dysregulated in fibroblasts collected from PD patients [43]. In order to study the direct effect of oxidative stress on the regulation of specific proteins in the presence of the LRRK2 R1628P risk variant, both primary fibroblast cells and cortical neurons were generated in parallel.

**Figure 3:**
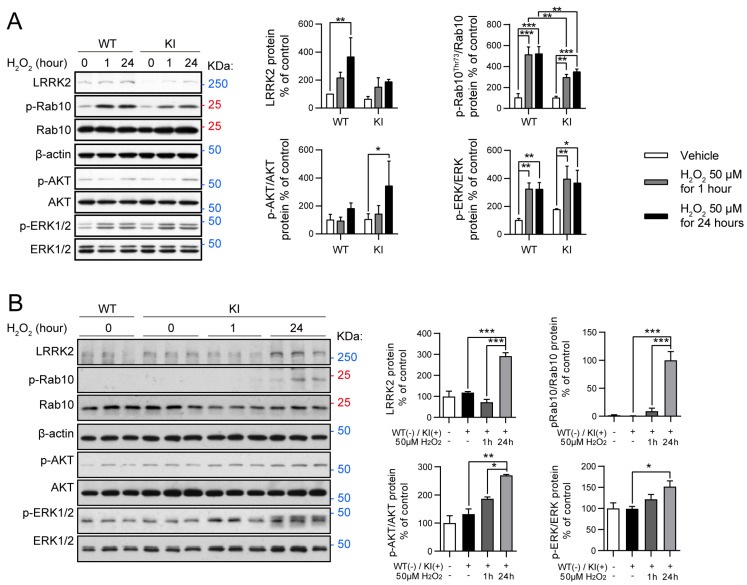
Oxidative stress modulates RAB10, AKT, and ERK activity in LRRK2 R1628P WT and KI primary fibroblast cells and primary cortical neurons. (**A**) Oxidative stress treatment. H_2_O_2_ was introduced to WT and KI primary fibroblast cells for 1 h (short-term) and 24 h (long-term). Subsequently, Western blot was performed using antibodies specific for LRRK2, p-RAB10, p-ERK, and p-AKT. Protein levels normalized to WT controls (mean ± SEM) (n = 3/group). (**B**) Western blot analysis of WT primary cortical neurons, KI primary cortical neurons, and KI primary cortical neurons that have been treated with H_2_O_2_ for 1 h (short-term) and 24 h (long-term). Antibodies specific for LRRK2, p-AKT, p-ERK, and p-RAB10 were used. Protein levels normalized to WT group (mean ± SEM) (n = 3/group). One-way ANOVA followed by Bonferroni post hoc comparisons, * *p* < 0.05, ** *p* < 0.01, *** *p* < 0.001.

Primary fibroblast cells of WT and KI mice were treated with either a vehicle (PBS), 50 μM H_2_O_2_ for 1 h (short-term treatment), or 50 μM H_2_O_2_ for 24 h (long-term treatment). Cells were then collected and key proteins related to the LRRK2 and the oxidative stress molecular pathways were analyzed (Figure 3A). Long-term H_2_O_2_ treatment induced a significantly increased expression of LRRK2 protein in LRRK2 R1628P WT primary fibroblast cells but not in LRRK2 R1628P KI cells (Figure 3A). RAB10 is a substrate of LRRK2 [43] and its phosphorylation is directly correlated with LRRK2 activity; importantly, p-RAB10 has been shown to be elevated in PD patients with the LRRK2 G2019S variant [44]. p-RAB10 was found to be significantly elevated after short-term and long-term H_2_O_2_ treatment in both LRRK2 R1628P WT and KI fibroblasts, with p-RAB10 expression being higher in LRRK2 R1628P WT than in LRRK2 R1628P KI cells a (Figure 3A, Supplementary Figure S1B). This suggests that the R1628P risk variant in mice induces inhibition of LRRK2 substrate activity in response to oxidative stress in primary fibroblast cells.

LRRK2 is a key regulator of the PI3K-AKT pathway which plays an important role in cellular growth and proliferation [45,46]. It should be noted that this molecular pathway has been implicated in neuroinflammation and *in vivo* studies have shown that inhibiting this pathway is protective against LPS-induced inflammation [47]. p-AKT was quantified against total AKT in the risk variant mouse line. While no significant change in p-AKT was observed after oxidative stress treatment in the LRRK2 R1628P WT primary fibroblast cells, an increase in p-AKT was detected in the LRRK2 R1628P KI cells after long-term H_2_O_2_ treatment (Figure 3A, Supplementary Figure S1B).

Lastly, the ERK/MAPK signalling pathway was investigated; this stress-activated pathway regulates cellular processes such as proliferation and differentiation as well as cell survival (by activating bax and subsequently causing apoptosis), and has been shown to be a downstream mediator of LRRK2 [48,49,50]. Dysfunction in the ERK/MAPK signalling pathway has been linked with PD [51]. Interestingly, ERK1/2 activity is known to be both neuroprotective and neurotoxic depending on the context, with transient activity being protective and sustained activity promoting cell death [52]. Significant increases in p-ERK (quantified against ERK) were found similarly in both LRRK2 R1628P WT and KI groups after both short-term (transient) and long-term (sustained) oxidative stress treatment (Figure 3A, Supplementary Figure S1B), suggesting that the risk variant has no direct effect on this molecular pathway.

Endoplasmic reticulum stress (ER stress), much like oxidative stress, has also been implicated in PD pathology [53]. ER stress activates the ER-associated degradation (ERAD) pathway which leads to target proteins being sent to the proteasome for degradation [54]. LRRK2 has been found to be a substrate for ERAD which hints towards the role of ER stress in LRRK2 parkinsonism [53]. In order to study the effects of ER stress, LRRK2 R1628P WT and KI primary fibroblast cells were treated with either a PBS vehicle or Thapsigargin (TG), a known ER stressor [55], for 1 h (short-term treatment) or 24 h (long-term treatment).

A significant increase in LRRK2 was observed in the LRRK2 R1628P WT group with short-term TG treatment, after which LRRK2 levels decreased considerably. This phenomenon was not present in the LRRK2 R1628P KI group which showed no significant change in LRRK2 levels after both short-term and long-term treatment (Supplementary Figure S1C). p-RAB10 quantified against RAB10, was significantly elevated after TG treatment in the LRRK2 R1628P WT primary fibroblast cells; this was not the case for the LRRK2 R1628P KI group. Expectedly, in correlation with the results obtained with the H_2_O_2_ treatment p-RAB10 levels were lower in the LRRK2 R1628P KI compared to the WT cells (Supplementary Figure S1C).

Eukaryotic initiation factor 2 alpha subunit (eIF2α) is a component of the ER stress pathway that inhibits global protein synthesis while enhancing the translation of stress-related genes when phosphorylated [56], such as GADD and CHOP. While GADD34 serves as a feedback loop to restore global protein synthesis, CHOP is involved in apoptotic processes [57]. The p-eIF2α levels, quantified against eIF2α to determine eIF2α activity, were increased upon short-term TG treatment but dropped back down after long-term TG treatment on both LRRK2 R1628P WT and KI primary fibroblast cells (Supplementary Figure S1C). Interestingly, GADD34 and CHOP levels increased drastically after long-term TG treatment in both LRRK2 R1628P WT and KI groups (Supplementary Figure S1C), suggesting that the translation of these proteins is a long but subsequent process of eIF2α activity level. In order to evaluate the level of programmed apoptosis following ER stress by TG treatment, cleaved caspase 12 (CC12) was quantified against caspase 12 (C12) [58]. Strikingly, there was no significant variation in CC12 levels across all groups, suggesting that neurodegeneration did not occur at this stage and on these cells (Supplementary Figure S1C).

In a parallel experiment, cortical neurons were isolated and cultured, and then exposed to the same H_2_O_2_ short and long-term stress treatments. Western blot was then performed on the four experimental groups. Proteins linked to LRRK2, and the PI3K-AKT and ERK/MAPK pathways were analyzed (Figure 3B). While there was no increase in LRRK2 after short-term oxidative stress treatment, a significant increase in LRRK2 was observed in the long-term oxidative stress treatment for KI cortical neurons (Figure 3B). A similar observation was made for p-RAB10 with the long-term oxidative stress group displaying elevated p-RAB10 levels compared to the vehicle and short-term oxidative stress groups (Figure 3B, Supplementary Figure S1D). These findings suggest that there was increased LRRK2 activity after 24 h of oxidative stress. Lastly, both p-AKT and p-ERK were found to be significantly increased in KI cortical neurons upon long-term oxidative stress treatment compared to the vehicle-treated KI neurons (Figure 3B, Supplementary Figure S1D). These findings hint towards oxidative stress increasing activity of both the PI3K-AKT and ERK/MAPK pathways in LRRK2 R1628P KI cortical neurons.

It should be noted that an increase in LRRK2 occurred as well but only in the long-term oxidative stress treatment, as if the effect was delayed. p-RAB10 increased eventually as well, and of great interest, both p-AKT and p-ERK were significantly increased in KI cortical neurons, especially upon long-term treatment. Taken together, these data show that the risk variant induces a higher sensitivity towards oxidative stress in cortical neurons.

### 2.4. RAB10, AKT, and ERK Activation Is Altered in Primary Midbrain Neurons from LRRK2 R1628P KI Mice

**Figure 4:**
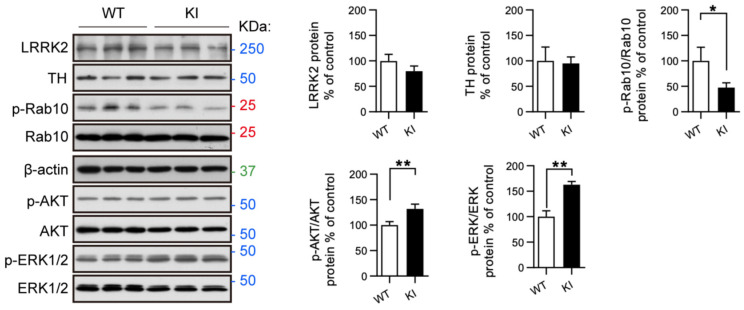
RAB10, AKT, and ERK activation is altered in primary midbrain neurons from LRRK2 R1628P KI mice. Western blot analysis of primary midbrain neurons from WT and KI mice. LRRK2, TH, p-RAB10, p-AKT, and p-ERK were analyzed. Protein levels normalized to WT controls, *t*-Test with Welch correction (mean ± SEM) (n = 3/group), * *p* < 0.05; ** *p* < 0.01.

Ventral midbrain dopaminergic neurons and cortical neurons were isolated from LRRK2 R1628P WT and KI pups and cultured; there were no obvious morphological differences between WT and KI cortical neurons (Supplementary Figure S2A). Next, key proteins related to TH, LRRK2, PI3K-AKT, and ERK/MAPK pathways were analyzed in midbrain dopaminergic neurons using Western blot (Figure 4). There was no apparent difference in the levels of LRRK2 and TH between WT and KI midbrain neurons (Figure 4). Although LRRK2 expression was similar, the LRRK2 substrate p-RAB10 was found to be significantly decreased in the LRRK2 R1628P KI group compared to the WT control group (Figure 4, Supplementary Figure S2B), echoing the results obtained in fibroblast cells challenged with H_2_O_2_ oxidative stress (where p-RAB10 was significantly less expressed in KI mice compared to WT mice). On the other hand, p-AKT and p-ERK were significantly elevated in the LRRK2 R1628P KI midbrain dopaminergic neurons indicating an increased activity of the PI3K-AKT and ERK/MAPK pathways (Figure 4, Supplementary Figure S2B). Overall, the LRRK2 R1628P risk variant in midbrain neurons seemed to increase PI3K-AKT and ERK/MAPK pathway activity and inhibit LRRK2 substrate activity.

## 3. Discussion

The LRRK2 R1628P risk variant has been found to double the risk of developing PD in humans [25]. It is largely found in East Asian populations and accounts for about 5 to 11% of PD cases in countries such as China, Singapore, and Taiwan [24]. Considering that this geographical region includes 22% of the global population, this risk variant can be considered of global interest [59]. However, it remains until today underexplored, mainly due to the lack of any *in vivo* mouse models. To address this, we generated a novel LRRK2 R1628P KI mouse model with the hope that it would mimic the disease observed in humans. Overall, these KI mice displayed cellular, molecular and behavioural features replicating human phenotypes that are predisposed to PD.

Key proteins involved in PD were dysregulated in adult LRRK2 R1628P KI mice. In particular, DAT was consistently decreased in KI mice (Figure 1C,D); DAT is localized in the presynaptic membrane of dopaminergic neurons and plays a role in transporting excess dopamine back into the neuron for recycling [60]. DAT dysfunction or decrease would therefore lead to increased levels of dopamine which was observed in the LRRK2 R1628P KI mice as well (Figure 1E). Another key feature of PD is the increase in α-synuclein aggregates, which was also observed in LRRK2 R1628P KI mice, providing further evidence that the LRRK2 R1628P KI adult mice display distinct PD phenotypes [61] (Figure 1C). Additionally, it is noteworthy that LRRK2 levels were increased in the striatum of LRRK2 R1628P KI mice but not the midbrain (Figure 1C); this may suggest that LRRK2 could accumulate in the striatum. In a future study, further analysis of the RNA expression may be performed to confirm this.

PD is characterized by motor dysfunctions; however, these symptoms are diagnosed rather late in the course of the disease. A first analysis of the LRRK2 R1628P mouse model could not allow us to detect such dysfunctions. Whether these defects were undetectable at this particular age, or whether KI mice would only display subtle deficits overall, the behaviour of these mice was not significantly modified. With the current growing hypothesis that a combination of genetic and environmental factors would increase susceptibility towards specific diseases, a longitudinal study was performed whereby WT and R1628P KI mice were subjected to stress consisting of neurotoxins injection at low doses (a mixture of paraquat and maneb) and subtle chronic psychological and physical stressors (UCMS, Figure 2A). Mice were treated at young adulthood and phenotyped at different ages. Interestingly, the activity of WT and KI mice measured in the open field and cylinder test decreased over the course of the study only when stressed. Stressed WT and KI mice exhibited movement and anxiety disorders with time, with the KI mice showing them earlier than the WT mice (Figure 2A). Additionally, only the Stressed-KI group displayed gastrointestinal dysfunctions, while the Stressed-WT group was largely unaffected (Figure 2D).

Since the combination of paraquat/maneb used in the stress treatment is known to trigger oxidative stress [36], we postulated that LRRK2 R1628P may confer susceptibility towards this particular stressor. An *ex vivo* model was thus employed to understand if and how oxidative stress impacts LRRK2 R1628P. Primary cultures of fibroblast cells were generated from WT and R1628P KI pups and subjected to a control treatment, short-term, or long-term oxidative stress. Culture extracts were analyzed and key proteins were quantified. As a result, p-RAB10 was significantly increased in the treated WT fibroblast cells as compared to treated KI cells (Figure 3A, Supplementary Figure S1B). Since p-RAB10 is a substrate of LRRK2 [62], it is reasonable to expect a greater increase in p-RAB10 with increased levels of LRRK2 (as is the case for the WT cells). Therefore, in LRRK2 R1628P KI primary fibroblast cells, oxidative stress attenuates LRRK2 substrate activity increase. This finding could be explained by the increase in LRRK2 after long-term oxidative stress in the WT cells which was not the case for long-term oxidative stress-treated KI cells (Figure 3A). However, it is still noteworthy that p-RAB10 is attenuated in the LRRK2 R1628P model. This is contrary to the present literature that shows that p-RAB10 is instead positively correlated with LRRK2 parkinsonism [44,62,63]. A more in-depth study of the mechanisms behind LRRK2 R1628P and its effect on LRRK2 kinase activity could shed light on this observation.

Additionally, while no significant change in p-AKT was observed after oxidative stress treatment in the WT group, p-AKT was increased in the long-term oxidative stress KI group which suggests increased activation of the PI3K-AKT pathway (Figure 3A, Supplementary Figure S1B). The PI3K-AKT pathway is involved in cellular growth, proliferation, and apoptosis [45] and it has also been implicated in PD neurodegeneration. Indeed, a few clinical findings indicate that AKT and p-AKT levels are reduced in the substantia nigra tissue from PD patients [64]. This pathway has been shown to act downstream of LRRK2 and is linked to oxidative stress by modulating mTOR, GSK-3, and FoxO3a, and its activation improves dopaminergic neuronal survival by repressing apoptosis (via the inhibition of BAD) [46,65]. Our study showed an activation of the PI3K-AKT pathway in oxidative stress-treated LRRK2 R1628P KI fibroblast cells. This may be due to compensatory mechanisms elicited by the LRRK2 R1628P risk variant which is absent in WT LRRK2. While oxidative stress differentially affected WT and R1628P KI fibroblast cells, ER stress largely led to similar outcomes in both WT and R1628P KI groups with fluctuations in p-eIF2α, GADD34, CHOP, CC12 being comparable throughout (Supplementary Figure S1C). This suggests that the LRRK2 R1628P risk variant plays a minimal role in the ER stress pathway.

With the knowledge that LRRK2 R1628P impacts the oxidative stress pathway, we next studied *ex vivo* cortical neurons in the presence of short-term and long-term oxidative stress treatment. Similar to treated primary LRRK2 R1628P fibroblast cells, LRRK2 R1628P KI cortical neurons displayed an increase in p-RAB10 after oxidative stress indicating increased LRRK2 substrate activity (Figure 3B, Supplementary Figure S1D). Once again, p-AKT was elevated with the H_2_O_2_ treatment showing increased PI3K-AKT pathway activation (Figure 3B, Supplementary Figure S1D). p-ERK was also found to be increased with H_2_O_2_ treatment; importantly ERK has been shown to promote neuronal cell death by activating bax and causing apoptosis [66].

Since the SNpc is located in the midbrain, primary midbrain neurons are of particular relevance when modelling PD under *ex vivo* conditions [67]. Primary midbrain neurons collected from WT and LRRK2 R1628P KI mice were analyzed to determine if there was any differential expression of proteins under naïve conditions (i.e., in the absence of oxidative stress). Interestingly, R1628P KI neurons were found to have reduced levels of p-RAB10 along with increased levels of p-AKT and p-ERK compared to the WT group (Figure 4, Supplementary Figure S2B), the latter being correlated with previous *in vitro* findings. Noteworthy, differences in transcription and translation between endogenous mouse *Lrrk2* and inserted human cDNA may exist, and a complete study would include the investigation of a mouse bearing the human wild-type *Lrrk2* under the same promoter.

In this study, we show *in vivo* and *in vitro* that the human LRRK2 R1628P risk variant triggers a susceptibility to stress, leading to the development of molecular and cellular dysregulations. *In vivo*, mice were stressed in adulthood; hence, the data report an effect of late stress in an organism that displays genetic susceptibility (none for the WT mice). While the genetic risk variant itself did not seem to trigger significant changes, the stress treatment affected both WT and R1628P KI mice, but at a late age. It is to be noted that the stress insult was not strong enough to induce an immediate response in contrast to what is observed in toxic-induced models for PD where the doses are significantly higher. However, KI mice were more susceptible and were affected to a greater extent, displaying earlier phenotypes as compared to their WT-stressed littermates; in other words, the course of the disease was accelerated.

Based on these findings, it is likely that environmental stressors can accelerate parkinsonism in patients harbouring the LRRK2 R1628P risk variant, more so than the general population, and especially when environmental insults start at the beginning of the organism’s development. Specifically, this study suggests that limiting exposure to the agricultural neurotoxins paraquat and maneb should be a vital public health measure against PD, particularly in East Asian agrarian populations where the LRRK2 R1628P risk variant is also prevalent [24]. Likewise, exposure to other oxidative stress-inducing agricultural toxins such as rotenone [68] should be restricted. Environmental factors have long been implicated in PD [69] and people bearing genetic susceptibilities may be prone to develop earlier or stronger phenotypes. Better understanding the link between genetic and epigenetic factors earlier in the course of the disease may present new therapeutic avenues to help delay LRRK2 R1628P parkinsonism.

The main limitation of this study is that only male mice were used. In order to minimize the influence of the estrous cycle on behaviour phenotypes, and to reduce the overall number of experimental groups and time points due to resource constraints, female mice were excluded. Nevertheless, sex-specific differences may be present in LRRK2 R1628P parkinsonism; therefore, it is important that sex-specific effects are characterized and evaluated in a future study.

In summary, the human LRRK2 R1628P KI mice display PD-like phenotypes combining behavioural and molecular features in response to different stress, characteristics that have not yet been described in an LRRK2 mouse model. This work provides an example of the complexity of the role of LRRK2 in PD while bringing to the community a new model that can be used to investigate further the balance between genetic and environmental factors in the development of the disease.

## 4. Methods and Materials

### 4.1. Animals

The mouse *Lrrk2* gene sequences were retrieved from the mouse chromosome 15 sequence from the Ensembl database, and the human *Lrrk2* (NM_198578.3) cDNA sequence was retrieved from the NCBI database. Mouse RP23-390I2 was used as the template for generating homology arms for the gene targeting vector, as well as the southern probes for screening targeting. The 5′ homology arm (~5.1 kb), human *Lrrk2* cDNA (~7.6 kb) and the 3′ homology arm (~3.5 kb) were generated by PCR. These fragments were cloned in the 3LoxP3NwCD or pCR4.0 vector, and were confirmed by restriction digestion and sequencing. Three stop codons were added to the 3′ end of the human *Lrrk2* cDNA to ensure proper translational stop. The final vector was obtained by standard molecular cloning. In addition to the homology arms and the knock-in region, the final vector also contained LoxP sequences flanking the Neo expression cassette (for positive selection of the electroporated ES cells), and a DTA expression cassette (for negative selection of the ES cells). The final vector was confirmed by both restriction digestion and end sequencing analysis (Supplementary Document S1). NotI was used for linearizing the final vector prior to electroporation.

Mice were generated by Taconic Biosciences (Cranbury, NJ, USA). Briefly, 30 μg of Notl-linearized final targeting vector DNA containing the R1628P risk variant form of the human LRRK2 gene (Figure 1A, Supplementary Document S1) was electroporated into 107 C57BL/6NTac ES cells and selected with 200 μg/mL G418. Two plates of resistant ES clones (around 192) were selected for screening by 3′ PCR. The selected clones were expanded, and an additional Southern confirmation analysis was performed to verify the homologous recombination with single Neo integration. Confirmed targeted clones that were further confirmed by PCR and sequencing were injected into blastocysts (B6Tyr) and male chimeras were generated. The male chimeras were further bred with wild-type C57BL/6NTac female mice. Heterozygous mice were identified from pups by PCR. The Neo cassette was removed by breeding the heterozygous to Cre deleters.

Animals were further bred under the same C57BL/6NTac genetic background (at least six backcrosses). The transgene segregated heterozygously and the genotype of the animals was determined by PCR on tail samples as advised by Taconic Biosciences, Cranbury, NJ, USA. Wild-type (*hLrrk2*(R1628P)^wt/wt^, or *mLrrk2*, herein named WT) non-carriers and homozygotes littermates mice for the *hLrrk2*(R1628P) mutation (*hLrrk2*(R1628P)^mut/mut^, herein named KI) were used for the experimental procedures while heterozygous mice (*hLrrk2*(R1628P)^wt/mut^, herein named HET) were kept for breeding purposes. Food and water were given *ad libitum*. Mice were ear notched and tested in all experiments in the same order according to their identification number (number/cage), following standard blocked randomization of testing and data processing. The experiments and analyses were always performed blind to the treatment and genotype. The study was carried out at the Animal Research Laboratory/Surgical Science and Research Laboratory located at Tan Tock Seng Hospital and co-managed by Tan Tock Seng Hospital and the National Neuroscience Institute, in strict accordance with the recommendations in the Guide for the Care and Use of Laboratory Animals of the National Neuroscience Institute and following the NACLAR guidelines (Ref TNI-06-04-006), in accordance with the National Institute of Health guidelines (USA).

### 4.2. Animal Treatment

Male mice were injected 2 times per week for 3 weeks (6 injections in total, i.p. at a dose of 10 mL/kg body weight) at 3 months of age with Paraquat (5 mg/kg in saline, Sigma Aldrich, St. Louis, MO, USA, #73488) and Maneb (15 mg/kg in saline, Sigma Aldrich, St. Louis, MO, USA, #45554) or saline (0.9% NaCl) [70,71] followed by a chronic stress protocol consisting of 6 continuous weeks of unpredictable chronic mild stress (UCMS) according to a semi-random 3-week schedule adapted from [72,73,74]. The paradigm included 1 to 3 stressful events per day and a regular check-up on the first day of each week where body weight and fur were assessed. Stressful events included: temporary transfer to (i) cages without sawdust (30 min to 3 h), (ii) cages filled with damp sawdust (2 h to overnight), (iii) cages filled with 1 to 3 cm water (5 to 10 min), (iv) cage tilted at 45° on either side (30 min to 3 h), (v) soiled cages filled with bedding from other mouse cages or from rat cages (1 to 3 h), (vi) exposure to reversed or dysregulated light/dark cycle (24 to 36 h), and (vii) social stress by putting 2 males together (1 to 2 h). Mice were stressed in social groups when possible (cage mates together) or isolated for the time of the stress only, allowing littermates to be kept together for several months in the facility for ageing. No water nor food restriction was applied.

For HPLC and immunoblotting, mice were euthanized by CO_2_ inhalation and cervical dislocation, and brain regions were collected, deep-frozen and stored at −80 °C until use. For immunohistochemistry, mice were anaesthetized with a mixture of Ketamine (Sigma Aldrich, St. Louis, MO, USA, 1867-66-9) and Xylazine (Sigma Aldrich, St. Louis, MO, USA, 7361-61-7) and perfused with a saline solution followed by cold paraformaldehyde 4%. Brains were then postfixed in the same solution for 48 h before being cryo-conserved with 30% sucrose in saline and kept at 4 °C until use.

### 4.3. HPLC Analysis

Samples were solubilized in 0.5 N perchloric acid. The levels of dopamine (DA) in the differentiated tissues were measured using a reversed-phase UltiMate 3000 HPLC System (Thermo Scientific, Waltham, MA, USA) with an electrochemical detector and a reversed-phase (Vydac Denali C18, 250 × 4.6 mm^2^, 5 μm, 120A pore size) and analyzed under the control of a Chromeleon^TM^ 7.2 Chromatography Data System. The mobile phase was a mixture of 1.3% Sodium acetate, 0.5% sodium 1-heptanesulfonate, 0.01% EDTA, 7% Acetonitrile (*v*/*v*) and 2% methanol (*v*/*v*), adjusted to pH 4.0 with pure acetic acid. All solutions for HPLC were double-filtered through 0.2 μm membranes and degassed before use. The flow rate was 1 mL per min. All chemicals were purchased from Sigma Aldrich, St. Louis, MO, USA.

### 4.4. Autoradiography Analysis

Autoradiography evaluation followed a previously published protocol [60]. Analysis was carried out on 10 μm thick midbrain sections. Autoradiography of DAT was performed using [^3^H]FE-PE2I as a radioligand. Sections were incubated for 15 min in 50 mM Tris–HCl buffer, pH 7.4, containing 120 mM NaCl, 5 mM KCl, 2 mM CaCl_2_ and 1 mM MgCl_2_. Next, incubations were carried out for 1 h in 50 mM Tris–HCl buffer, pH 7.4, containing 120 mM NaCl, 5 mM KCl, 2 mM CaCl_2_, 1 mM MgCl_2_ and 1 nM [^3^H]FE-PE2I. Non-specific binding was determined in neighbouring sections in the presence of 10 μM GBR12909. Sections were washed 3 times for 3 min in cold 50 mM Tris–HCl buffer, pH 7.4, followed by washing in distilled water. Radioactivity was quantified using phosphor imaging (scanner: Fuji BAS-5000 image reader: imaging plates: BAS-TR2025, Fujifilm, Tokyo, Japan). The measured photo-stimulated luminescence (PSL)/mm^2^ values were converted into radioactivity units and then into binding density (fmol/g) based on intensity values obtained using tritium standards (Microscales, American Radiolabeled Chemicals Inc., St. Louis, MO, USA). Specific binding was calculated by subtracting nonspecific binding from the total [^3^H]FE-PE2I binding.

### 4.5. Immunohistochemistry

Brains were cut coronally at 30 μm with a microtome and sections were collected in series of 8 in an anti-freeze solution (30% Glycerol, 30% Ethylene Glycol, 40% 0.1M PBS, Sigma Aldrich, St. Louis, MO, USA) and kept at −20 °C until staining. Each series was stained following an immunostaining protocol for free-floating sections, as described in [75]. Briefly, sections were washed in PBS and endogenous peroxidase activity was blocked by a 20 min incubation in 2% H_2_O_2_ in 10% methanol in PBS. After extensive washing in PBS, samples were pre-incubated for 1 h with 10% horse serum to decrease the background, then incubated overnight in anti-Tyrosine Hydroxylase (Rabbit anti-TH, Novus Biologicals, Centennial, CO, USA, #NB300-109, 1:500 in 5% serum in PBS). Sections were washed in PBS and a second biotinylated antibody was used before washing and revealing the staining with DAB (VectorLabs, Newark, CA, USA, VECTASTAIN Elite ABC Kit #PK-6101). Mounted slides were observed with a Zeiss (Oberkochen, Germany) microscope and the number of TH positive cells was quantified by an unbiased stereological method by means of the Stereologer 2000 software (version 2.1) from Stereological Resources Centre, Chester, MD, USA. Briefly, optical fractionator frames were distributed randomly across the reference space and each frame, viewed under 63× oil immersion lens and was counted for dark-stained somas using a *z*-axis dissector probe (frame area 25; height 10; guard height 2; frame spacing 100). All counts were performed blinded to the mouse genotype and treatment.

### 4.6. Cell Culture

Primary neuronal culturing followed a previously published protocol [76]. Primary ventral midbrain neurons and cortical neurons were isolated from E15.5 LRRK2 R1628P WT and KI mouse midbrain and cortex, respectively. The midbrain and cortex were dissociated in Hepes-buffered Hanks’ balanced salt solution (HBSS), incubated in 0.25% trypsin (#32777-44, Nacalai Tesque, Kyoto, Japan) for 20 min (midbrain) or 45 min (cortex). They were subsequently washed with seeding medium [Dulbecco’s modified Eagle’s medium (DMEM; #D1152, Sigma Aldrich, St. Louis, MO, USA) with 5% fetal bovine serum (FBS; #10500-064, Gibco, Waltham, MA, USA)] and 1× HBSS (#14185-052, Gibco, Waltham, MA, USA). Brain tissue was triturated using a fire-polished glass Pasteur pipette in culture medium [neurobasal medium supplemented with 2% B27, 1% penicillin-streptomycin (PS; #15070063, Gibco, Waltham, MA, USA), and 1% L-GlutaMAX (#35050-061, Gibco, Waltham, MA, USA)]. Cells were allowed to settle for 2 min. Dissociated cells were counted and seeded in cell culture plates coated with 0.001% poly-L-lycine (#P4832, Sigma Aldrich, St. Louis, MO, USA).

Fibroblast cultures were obtained by collecting the whole abdomen from E15.5 LRRK2 R1628P WT and KI embryos. The tissue was digested in Hepes-buffered Hanks’ balanced salt solution (HBSS), incubated in 0.25% trypsin (#32777-44, Nacalai Tesque, Kyoto, Japan) for 45 min, and washed with seeding medium [Dulbecco’s modified Eagle’s medium (DMEM; #D1152, Sigma Aldrich, St. Louis, MO, USA) with 5% fetal bovine serum (FBS; #10500-064, Gibco, Waltham, MA, USA)] and 1× HBSS (#14185-052, Gibco, Waltham, MA, USA). Tissue was triturated using a fire-polished glass Pasteur pipette in culture medium [Dulbecco’s modified Eagle’s medium (DMEM; #D1152, Sigma-Aldrich) with 10% fetal bovine serum (FBS; #10500-064, Gibco, Waltham, MA, USA), 1% penicillin-streptomycin (PS; #15070063, Gibco, Waltham, MA, USA), and 1% L-GlutaMAX (#35050-061, Gibco, Waltham, MA, USA)]. Cells were allowed to settle for 1 min. Cells were seeded in uncoated culture plates.

For oxidative stress treatment, 50 μM H_2_O_2_ (#32338, Sigma Aldrich, St. Louis, MO, USA) was added to culture media for 1 h (short-term treatment) or 24 h (long-term treatment) [77,78], dosage and duration were further validated in primary neurons which show increased cleaved caspase 3 (CC3) with H_2_O_2_ treatment (Supplementary Figure S1A). For ER stress treatment, 1 μM Thapsigargin (HY-13433, MedChemExpress, Monmouth Junction, NJ, USA) was added to culture media for 1 h (short-term treatment) or 24 h (long-term treatment).

### 4.7. Behavioural Testing

Mice were assessed mostly as described in [79]. Briefly, animals were tested at 6, 9, 12, and 16 months old. Test–retest occurred every 3 months only, a sufficient amount of time to minimize confounding effects from experimenter handling and habituation to testing. On the day of testing, mice were brought to the experimental room about 20 min before for acclimatization and were tested within 3 h between 9:00 a.m. and 12:00 a.m. The experimenter was blinded to the treatment of the mice during the testing, sampling and collection of the data. The following 3 procedures were subsequently performed in this order and with at least 24 h rest between each one of them:

Open field: Mice were placed in the centre of a grey arena (45 × 45 × 40 cm^3^) and allowed to explore for 15 min in a dimmed environment (approximately 50 Lux). The test was video-recorded and the animals were tracked for their locomotion, grooming, rearing, and exploring behaviour with ANY-maze (Stoelting, Wood Dale, IL, USA).

Cylinder test: Mice were put in a transparent Plexiglas cylinder (12 cm diameter, 25 cm high) for 5 min and video recorded. The total number of rearing was scored from the videos by a blind experimenter using the key-in setting of ANY-maze.

One-hour stool collection: Mice were transferred to an empty cage and stool was collected for 1 h. The faeces were collected as soon as possible in sealed Eppendorf tubes. The tubes were weighed and then water evaporated overnight to note the dry weight of the stool 12 h later.

### 4.8. Protein Extraction and Western Blotting

Tissue and cell samples were first solubilized with a Pestle Motor (VWR, Radnor, PA, USA) in an N-PER Neuronal Protein Extraction Reagent (Thermo Scientific, Waltham, MA, USA, #87792). Lysates were then centrifuged at 4 °C (13,200× *g* for 30 min). Supernatants were collected and the proteins samples were resolved using SDS-PAGE (6%, 8%, 13.5%); Proteins were transferred to polyvinylidene difluoride membranes (Merck Millipore, Burlington, MA, USA, #IPVH00010) and blocked with TBST (tris-buffered saline-tween 20) supplemented with 3% BSA (Bovine Serum Albumin) (Sigma Aldrich, St. Louis, MO, USA, #A5611). The primary antibodies used were LRRK2 (Sigma Aldrich, St. Louis, MO, USA, HPA 014293), DAT (Santa Cruz Biotechnology, #sc-14002), Dopamine D2 Receptor (Merck Millipore, Burlington, MA, USA, #AB5084P), β-Actin (Abcam, Cambridge, UK, #ab6276, AC15), α-synuclein (Sigma Aldrich, St. Louis, MO, USA, #S3062), pThr73 Rab10 (Abcam, Cambridge, UK, #ab230261), Rab10 (Cell Signaling Technology, Danvers, MA, USA, #8127), Tyrosine Hydroxylase (Sigma Aldrich, St. Louis, MO, USA, #MAB318), pAkt (Ser473) (Cell Signaling Technology, Danvers, MA, USA, #9271), Akt (Cell Signaling Technology, Danvers, MA, USA, #9272), pErk1/2 (Thr202/Tyr204) (Cell Signaling Technology, Danvers, MA, USA, #9101), and Erk1/2 (Cell Signaling Technology, Danvers, MA, USA, #9102). Rabbit anti-mouse (GE Healthcare, Chicago, IL, USA, #NA934V), or mouse anti-rabbit (GE Healthcare, Chicago, IL, USA, #NA931V) were used to react with the corresponding antibodies. Bands were visualized by enhanced chemiluminescence (Thermo Scientific, Waltham, MA, USA, #32106). Densitometry analysis on the bands was calculated using ImageJ software, version 1.53a.

### 4.9. Data Analysis and Statistics

In the behavioural part of the study, data coming from either the automated analysis (in the open field) or manual recordings was analysed statistically (IBM SPSS Statistics version 25.0) according to the number of factors (animal number, age, genotype, behaviour recorded, repeated measures) and adequate statistical test (Welch’s *t*-test for equality of variances or ANOVA followed by post-hoc comparisons accordingly). Data were expressed as Means ± Standard Error of the Means. Body weight was considered a covariate in the analysis of behavioural experiments. In the biochemical analysis, data was expressed by Means ± Standard Error of the Means. Statistical tests are described in the legend according to the data shown.

## Data Availability

All data generated and analyzed during the current study are available.

## Supplementary Materials

The following supporting information can be downloaded at: https://www.mdpi.com/article/10.3390/ijms26083556/s1.

## Abbreviations

ARG: Autoradiography; CC3: cleaved caspase 3; CC12: cleaved caspase 12; DA: Dopamine; DAT: Dopamine transporter; DD2R: Dopamine D2 receptor; eIF2α: Eukaryotic initiation factor 2 alpha subunit; ER: endoplasmic reticulum; ERAD: endoplasmic reticulum associated degradation; HPLC: High performance liquid chromatography; KI: Knock-in; LRRK2: Leucine rich repeat kinase 2; PCR: Polymerase chain reaction; PD: Parkinson’s disease; PQMB: Paraquat/Maneb; ROS: reactive oxygen species; SN: Substantia nigra; SNpc: Substantia nigra pars compacta; TG: Thapsigargin; TH: Tyrosine hydroxylase; UCMS: unpredictable chronic mild stress; WT: Wild-type.
