# Supplementary material for: Environmental Factors Exacerbate Parkinsonian Phenotypes in an Asian-Specific Knock-In LRRK2 Risk Variant in Mice
Source: Int J Mol Sci. 2025 Apr 10;26(8):3556. doi: 10.3390/ijms26083556 (PMC12027425; doi:10.3390/ijms26083556)
Supplement: Supplementary file 1 [file ijms-26-03556-s001.zip › ijms-3548680-supplementary.pdf]

Supplementary Figure S1

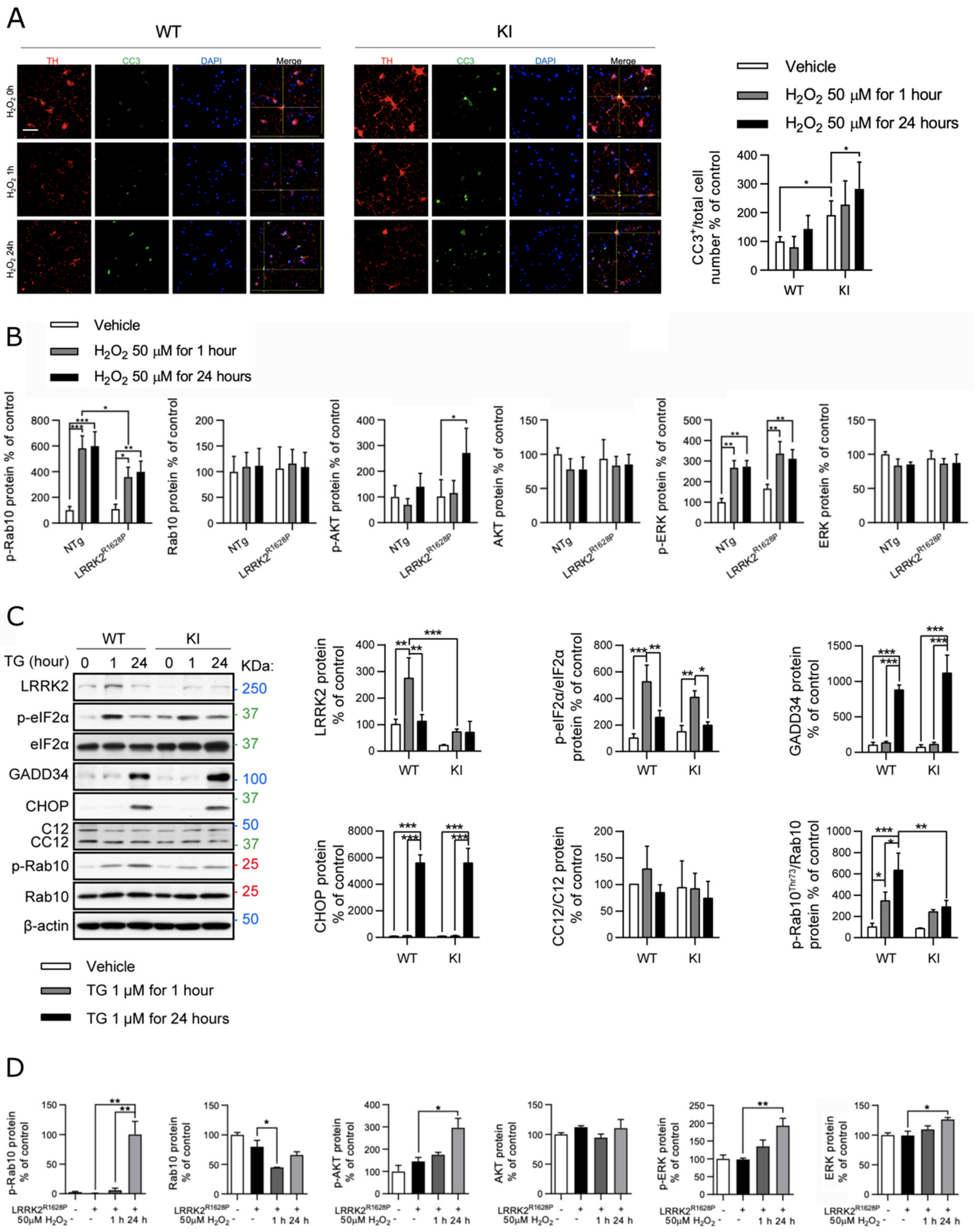

Supplementary Figure S1. Oxidative stress protocol validation and TG treatment. (A) Oxidative stress treatment validation. H_2O_2 was introduced to WT and KI primary midbrain neurons for 1 hour (short term) and 24 hours (long term). Representative images under 40X magnification. Red staining indicates TH, Green indicates CC3, Blue indicates DAPI, scale bar: 50 μm. **(B)** Oxidative stress treatment. H_2O_2 was introduced to WT and KI primary fibroblast cells for 1 hour (short term) and 24 hours (long term). Western blot was performed for p-RAB10, RAB10, p-AKT, AKT, p-ERK, and ERK.

Protein levels normalized to WT controls (mean ± SEM) (n=3/group). **(C)** Endoplasmic reticulum stress pathway is largely unaffected by LRRK2 R1628P KI. TG was introduced to WT and KI primary fibroblast cells for 1 hour (short term) and 24 hours (long term). Next, western blot was performed using antibodies specific for LRRK2, p-eIF2α, GADD34, CHOP, CC12, and p-RAB10. Protein levels normalized to aged-matched WT controls (mean ± SEM) (n=3/group). **(D)** Oxidative stress treatment. H_2O_2 was introduced to WT and KI primary cortical cells for 1 hour (short term) and 24 hours (long term). Western blot was performed for p-RAB10, RAB10, p-AKT, AKT, p-ERK, and ERK. Protein levels normalized to WT controls (mean ± SEM) (n=3/group).

Supplementary Figure S2

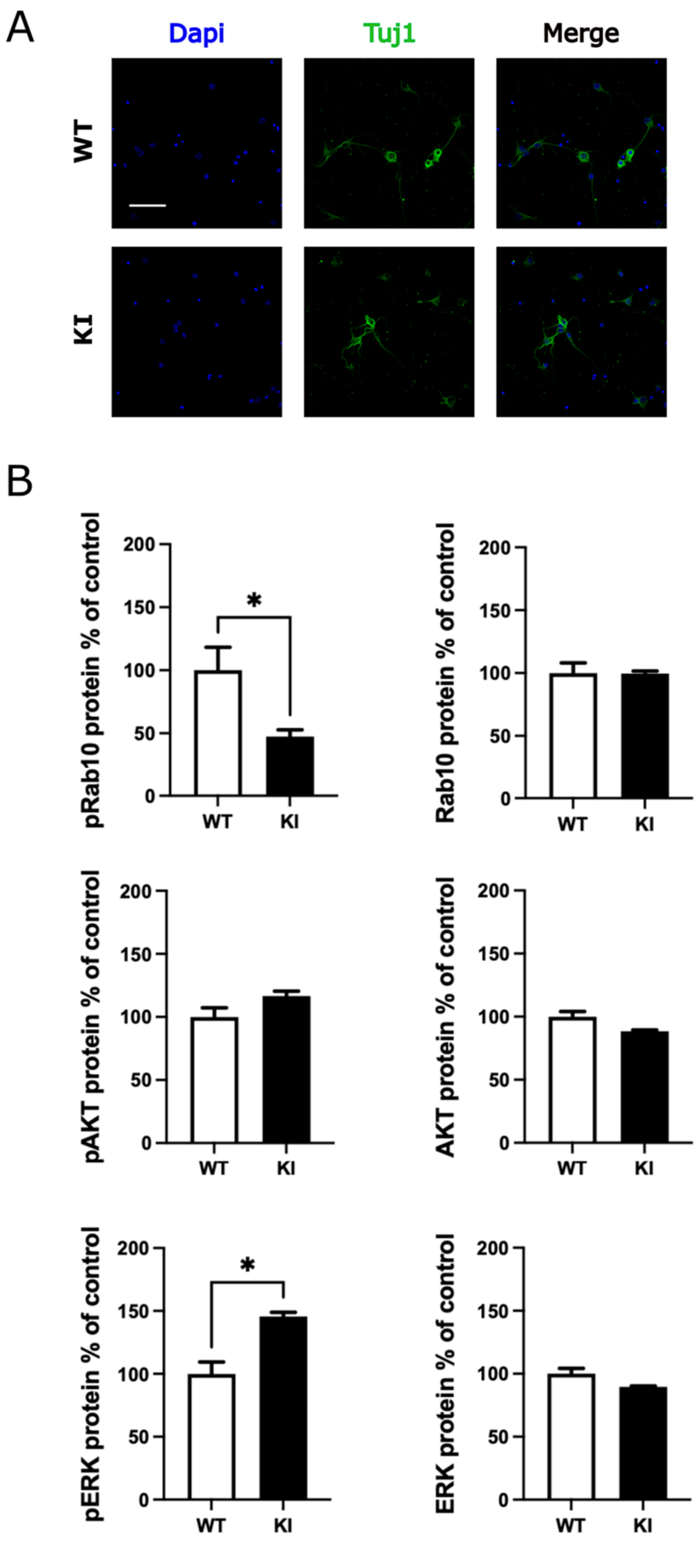

Supplementary Figure S2. RAB10, AKT, and ERK activation is altered in primary neurons from LRRK2 R1628P KI mice. (A) Representative images of cortical neurons under 40X magnification. Blue staining indicates DAPI, Green indicates Tuj1, scale bar: 50 µm. **(B)** Western blot analysis of primary midbrain neurons collected and cultured from WT and KI mice. p-RAB10, RAB10, p-AKT, AKT, p-ERK, and ERK were analyzed. Protein levels normalized to WT controls (mean ± SEM) (n=3/group).

Supplementary Document S1

Final Vector Map for Lrrk2 (R1628P)

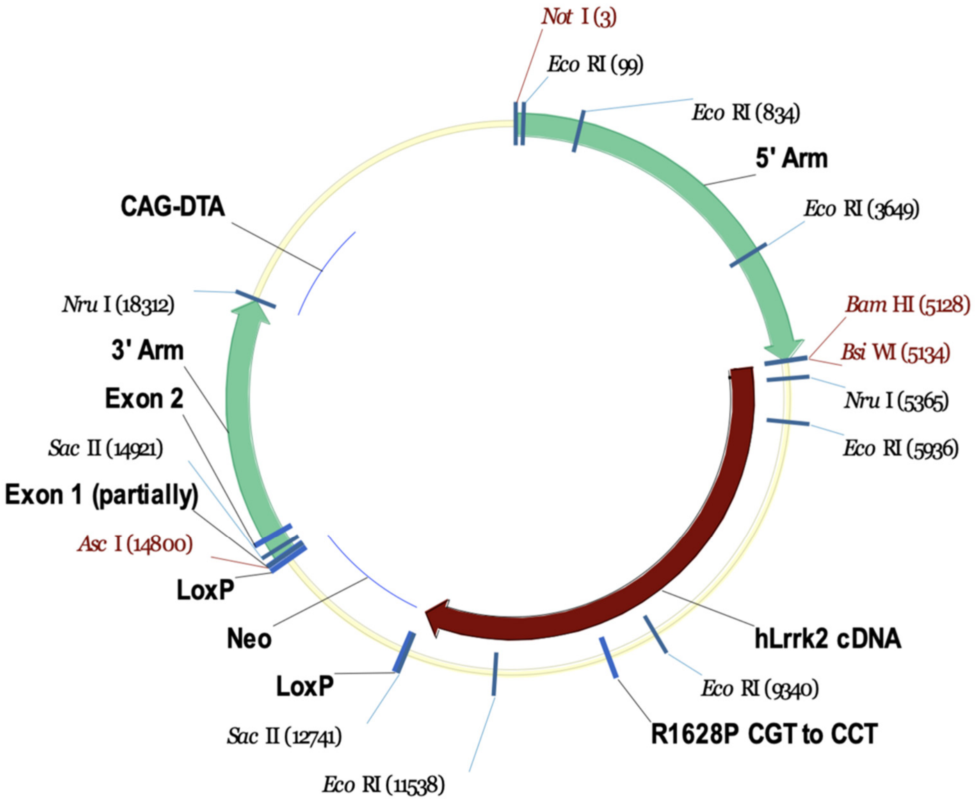

Final Vector Sequence for Lrrk2 (R1628P)

Homology arms in green, knock in region in red. R1628P mutation in blue. LoxP sites underlined. Confirmed sequences highlighted.

```
  1 GCGGCCGCTG GAGGAAACAT GGGAGAGAGG GTTTCTCACT TACTGTTGGT GGGCGGGTTT
 61 GTCCAGCTGG TGCAGCCACT GTGGAAATTG CTGTGGAGAA TTCTCAAAAA TCTAAAACTG
121 AATCTACCAT CGGACCCACC CATAACTCTC CTTCCCCTAT GCCTGAAGGA CTTGACATCA
181 GTATTTTTCC CAAACCATGT ATTGCTACTC TGTTTATAAT AGATAGGAAA TGGAAACAAA
241 ATAAATGTCC TCCAACCAAC AACTGAAAGA ATGAAAATAC AAACCATGGG ATACTATTCA
301 GCCTTAAAGA CAGATAAAAG TATGAACTTT GTAGATAAAT GGATGGAACT CAAAAAGATA
361 ATACCTACTG TGGTAGCCTG GACACAGAAA GACAAATATC ATTTGTTCTG TCAAATCAGA
421 GACTTTTAGC CTCTAATCTC TAGATGTAAG TACATACCCT GGAGTAACTG TAGAATCCAG
481 GAAAGTGCAA AGGGGCCATT GCTGAGGGAG GGGTTGAGCA GAAGCTGTAG AGAGGGAATG
541 TCAGGAAATG ATTGGGGACG TGGAAAACAA GAAGGGCTTT AATTAGGAAG TGGGGAGATA
601 AATACAGATG AAGCATGGTA AAATAAGAAA TATATCTGAA GAAGTAATAA GGAACCATTT
661 ATTAATTACC TGAAAAAGGA AACCCTATTA TACACATAAG TTTGTGTGTA AGTATATATT
721 TATAGTTCAA ATGAAATCGT TTTTTGTCTA GGCTGACAAT GCTGTCCAAG AGACTCCAAG
781 AACACACAAG GCTATGGCTA TTGTCCTTGG TTTCTCCCAA GATACGGAAG GTGAATTCCT
```

841 ATGGCTGAGG ATAATGTATT TCAGAGGCAG AACCCAGAGA CCCCTGCCTG GAACTGACAT
901 AAATGCCTCC ACTCTCAGGA CTAGCTGATA TGGTATCAGA AGGTGCCATG CAAGCTTCCA
961 GTGCAAAGAC GTTGTCAACA GTCCCTCTTA CTTATGATGC TTATAAACTT AAACAGAGAT
1021 GAGCATGGTG TGATCACCCC AAGTCTTCCG CAGTTGCATG GACACCATTT TGGTAAACAA
1081 CTCTCTAATT GGATTTAAGA ATCACTCAAC AAGAGGCAAA CCATGCCTGT TACCAGAAAC
1141 TGAGCCAACT ACCCAATGCT AATGAAGCCA TGGATTTTGG AGGAGAGTAT ACAACCACTA
1201 CTTCACTAAA CAAGCATGAC CCCTAACTAC ATTCTTAATA TTTGTCCTCA TATTCCCAGG
1261 TAACTGTAGT CTTTCAGCCC CTCATTGAGG AAACTTCTCT TTGTACCAGA TTTGGACTAT
1321 TACAGAAAAC AACAGACAAT CAAAATGTAC AGTTATGAAT CTTAGTTGTT ATGGATACAT
1381 CTATAAAACA CATTGGAACC TAAGGCTGAG GGAACATTGT GTAAGAAGTA GAAAGATTGT
1441 AGTTGCCAGA TCAAGGACTT TGCTGTGAGA TTGTACCTCC TAGTAAAGTC AAAAGGTCTC
1501 ATCAACAAAA CTGCCTGAAC AAAGACAGCA TTGTCTAATG ACAAAGTACA TGAGGGAAAG
1561 ACCACAGGCC TTAAGTCTGT AAAAAGAATT ATAGGCAAAT AAGGAATGCT GAAATAAGAG
1621 AAAGAGTTTT ACTTGGGGAA GATGCACAAA AAAATTGGTT GTCCAGTACC AAATAGTCGG
1681 CTACGAAAAT ATGCATACAC CAGGCAGTGG TGGCGCACAG CTTTAATCCC AGCACTTGGG
1741 AGGCAGAGGC AGGCGGATTT CTGAGTTCGA GGCCAGCCTG GTCTACAGAG TGAGTTCCAG
1801 GACAACCAGG GCTACACAGA TAAACCCTGT CTCAAAAAAA AAAACCAAAA TACAAAAACA
1861 TAAATAGAAA AAAAAAAGAA AATATGCATA CAAGTAACAT TGTACAGACT GAGAAGGTTA
1921 TATTTAGGAA TATATTATTA GAATAATAAT TAGGGATAAT ACATTATCAT GCTAAATATT
1981 GTTCTCTCTC TCTCTCTCTC TCTATATATA TATATATATA TATATATATA TATATATATT
2041 ATCACACACA CACACTTATT TCTCACATAT CCTCCCATCT GTGTAATACT GTATACTGTG
2101 TTACTCTTTC CCTGGCTCTT TATCCAAATA ATTTGATGAT AAAAGTAAGC TTCATCTATT
2161 GTACACACTG TAGCTTATGT AAGGATTTCA GGATGGAATG TGTGGCACCT CATATGGAAA
2221 TCTAACTTGG TGTGCTCTTT GTGTCTGTTA GTAAAGAAAA CACCTGTTCC TGATTCACAG
2281 GAAGTCACTA GGAGCTGGAT AAACCGTGCT TCCTGGGCCT GTCCCCTCGG TGACCCCAGT
2341 GGTACTGTTT CTTCCTCTGG AGAAGAGATA CACAGAGGAT ACTGTGTCCC TGAATCATGG
2401 CTGTTTGAAC AATTCTTTTA CCATAAGTGC CAGGGCTATT CTAACCTTTA GGACCTTCTG
2461 CACTATAGAT GGATGTGATT CATGTTATAT TTTTGTTACT GTTAAGAGAC AGTATCTTTT
2521 TATATTAAAT CTTCCTATTT ATTACTCCTA TTATTTTTCT CATTTATTTG TGTGTTTTAT
2581 GAGTTTGATC ATTTCTTTGC AAAAGTTTTG TATATCTGTA TGTTTAGACT TATCCCCATT
2641 CTTTTTCTCA TTGTTGATTC ACACACACAA TTGGACTCAT CTTCCTGAGC ATATTCTTTA
2701 TAGAGATGAG CATAGTTTTC TTAGACTTCC TGCTCATTTA CAGTAAACTT ACATTTTCCT
2761 CGAAATGCCT GTTGGGGTGC TGCTGTCTCT CCAGTGACAT ATTAAAAACT CTTCAAGTTA
2821 ATTGGTTCTT CCATATGAAA GCCCCAGCCA TTTACCATTT TCCTTTTGTT TACTTTTTCT
2881 TTCTTCTGAT GGCAGAATAT CTGAGTTTTA ATGAATCACA AATGTTAATG TTGTGTGTCT
2941 TCTGTCACTG CGTTTGCTTT TAATGCTGGG CTAAATAACG TTTACTATCC AGTGCCTCCT
3001 AATTATTTTT TATTTGCTTT TTAACCCATC TGTAATTTGT TTTGGTGTTT GCTGTGGTTG
3061 GCCATGTCAG TTTTATTTCT TTCTTCCTCA GAAGTTAGGT AAACATTGAG TGTTATTTTA
3121 CTATTCTTTG ACTACTTAGT TATGAACTTC TGTCTGCAGC CATCATCTTC ATTCTCGTTA
3181 ACTGGGAAGT TAATGAGTCT TGGAAATTTA GTCAATCAAT TCATCACATT TAAAACTCAA
3241 GGACACTTTG CCCTAAGGTG TACTGGCTTC TGGCAAATGT TTCCAGCAGA GAGCTCAAAG
3301 TACACCCTGT TTGGTGCCCT GCTGAGTATG GAGTCCTACA GGTGGATGAT AGTCCTACAA
3361 GGGGACTTTG GAAACACGGT GTCACTTAGA ATTTTCTTTT GAAGGGACAT AAGTCTCAAT
3421 TTCTGGCAAG GTTTTTGGTG TGTGTGTGTG TGTGTGTGTG TGTGTGTGTG TGTGTGTGTG
3481 TGTGTTTTAA CAAGAAGTAA ATAAAATACG GGGGAAAAAA GAAAAGAAAA TCTGAGGTCC
3541 TCATGTCATG AAAGCGCATA GAAGAGGTAA TATAGGTCTC ATATATGTCA TCAGCTGAGA
3601 GTTTTGTCAT TATGGAAAAT TACACACAGA GAGGAGCAAT GCCCCAGGAA TTCATCTCTT

3661 ACCTTCCACA ATTCCCAGTT CATTTCATGT CTAATTCTTC CCGTTTTGAC CCCACCCTTC
3721 AATCGTGGGT TACTTGGAAG CAAATCCTAA CTATTCTAGC CTTGCAAATA TTAGCACTTT
3781 ACTACCACGC AAAGGTGAAA ACCCTTGGCA ACAAGAAGCA CTCAGTGAGT ACCCAAGCTG
3841 ATGGAAACTT GCTTTCTTGT TCTGCTGAAA GTGCAGAGTG TGGACAAACT TTCAGCCTGA
3901 CCTTCAGTTG TGTCTTGGCT CTCAACCCGC CTCTCAAACC CCTCCACCTC TTACCCATCC
3961 CAAAGACATT TCCTCAGACA GGAAAGAACT CCTCATCCGT TTGTGAATTG TTATCAGAAT
4021 GTTAACGAAC TAGAAGCCAG GAAATAAATT AACTGCTCTA ATTCTCCTTC GGGGTAAATA
4081 ACCTTGAGGT TGGGTCATCT GAGACCATAG AACATCCAAG CCTTGACTAC AGCACTGTAC
4141 CAGAGACTTC TCTTCTTGAA AAACCTTACT ACGTGAGCAT TTTCTTTTGC TTTCTTCTTG
4201 GGATGCAACA TCTTCTTCAC AGGGATGCTT TAGCACTGTG CCTTGCAGAT CTGTCCCCTT
4261 CTCCCCCGCC CCCTCCCCTT TCTTCTCCTG CTTCAACTCT TCATTTACCT CTTCTCCTTC
4321 CCCCTCCCCC TCTCCCCCTG CTCTCTCCCT CTCTCTTCTT CCTCTACTTC TTCCTCCCTC
4381 TCCTTTTCCC TTCCCTTTCT CCCCTCCCCC CATTCCTTCT TTCTAGGTAG GCTCTTATAG
4441 CCCAGGCTGG CCTTGAACTT GTATGAAGCA AGGATGGCCT CCGATTTCTT TGGTTCTTGT
4501 CTTCACCTCC CAGGTGCTGG AATTAGCAGT TGCCCCGGCT TTCAGAATAC CCCTTTTAAC
4561 TTTAGAGGCC CGGGCAGCAG TACTAATAAC TAGTTCAGAC CAAGAATGCC GTTTAAAGAC
4621 TGTTTCTTCC AACCTTAGCT TCACTGACTC TGCTGTGAGT TCCATCCTTG AGAGCTGAAT
4681 CAGAGCTCCC AGATCCTGCC TGCAAACTCA AAACAGAGAG AGGCCTTTTG GCAACCACAA
4741 CCAGAGCTCA GGATAACCAA CAAGCAAACA ACTCCTAAAG CCCATGTAGC TGGCCGATTT
4801 TGATTGTTAA ACGCTGCATT CTTTGGGGAG CAAATTACTC CTCTCCCTAT AGGGGGAACT
4861 CTTAAAAGAA ACCGCTTTCC TGAAAGGGGC CAACTGGGCG GTGTCCTCCT TTCCCAACGC
4921 CCCTTTGCTA TTCTTAGTGG CGGGGTGGCG GCTTTCGCTT TTCCCCGGCG GAGTTCCCAG
4981 CGGCGCGATG CGGCTGGGCA GGCCGGGCTG AGACTGGGCA GGGGCGGTCG CCGTCACCGT
5041 CTGGGCGCGG CTGGGGACCG AGGGCTGGGC CTGCAGCGGA GGCGGCCGAG CAGCTCTGAG
5101 AGCAGGAGCC GTCCCAGCTC GCCGCAGGAT CCCGTACGAC CATGGCTAGT GGCAGCTGTC
5161 AGGGGTGCGA AGAGGACGAG GAAACTCTGA AGAAGTTGAT AGTCAGGCTG AACAATGTCC
5221 AGGAAGGAAA ACAGATAGAA ACGCTGGTCC AAATCCTGGA GGATCTGCTG GTGTTCACGT
5281 ACTCCGAGCA CGCCTCCAAG TTATTTCAAG GCAAAAATAT CCATGTGCCT CTGTTGATCG
5341 TCTTGGACTC CTATATGAGA GTCGCGAGTG TGCAGCAGGT GGGTTGGTCA CTTCTGTGCA
5401 AATTAATAGA AGTCTGTCCA GGTACAATGC AAAGCTTAAT GGGACCCCAG GATGTTGGAA
5461 ATGATTGGGA AGTCCTTGGT GTTCACCAAT TGATTCTTAA AATGCTAACA GTTCATAATG
5521 CCAGTGTAAA CTTGTCAGTG ATTGGACTGA AGACCTTAGA TCTCCTCCTA ACTTCAGGTA
5581 AAATCACCTT GCTGATATTG GATGAAGAAA GTGATATTTT CATGTTAATT TTTGATGCCA
5641 TGCACTCATT TCCAGCCAAT GATGAAGTCC AGAAACTTGG ATGCAAAGCT TTACATGTGC
5701 TGTTTGAGAG AGTCTCAGAG GAGCAACTGA CTGAATTTGT TGAGAACAAA GATTATATGA
5761 TATTGTTAAG TGCGTTAACA AATTTTAAAG ATGAAGAGGA AATTGTGCTT CATGTGCTGC
5821 ATTGTTTACA TTCCCTAGCG ATTCCTTGCA ATAATGTGGA AGTCCTCATG AGTGGCAATG
5881 TCAGGTGTTA TAATATTGTG GTGGAAGCTA TGAAAGCATT CCCTATGAGT GAAAGAATTC
5941 AAGAAGTGAG TTGCTGTTTG CTCCATAGGC TTACATTAGG TAATTTTTTC AATATCCTGG
6001 TATTAAACGA AGTCCATGAG TTTGTGGTGA AAGCTGTGCA GCAGTACCCA GAGAATGCAG
6061 CATTGCAGAT CTCAGCGCTC AGCTGTTTGG CCCTCCTCAC TGAGACTATT TTCTTAAATC
6121 AAGATTTAGA GGAAAAGAAT GAGAATCAAG AGAATGATGA TGAGGGGGAA GAAGATAAAT
6181 TGTTTTGGCT GGAAGCCTGT TACAAAGCAT TAACGTGGCA TAGAAAGAAC AAGCACGTGC
6241 AGGAGGCCGC ATGCTGGGCA CTAAATAATC TCCTTATGTA CCAAAACAGT TTACATGAGA
6301 AGATTGGAGA TGAAGATGGC CATTTCCCAG CTCATAGGGA AGTGATGCTC TCCATGCTGA
6361 TGCATTCTTC ATCAAAGGAA GTTTTCCAGG CATCTGCGAA TGCATTGTCA ACTCTCTTAG
6421 AACAAAATGT TAATTTCAGA AAAATACTGT TATCAAAAGG AATACACCTG AATGTTTTGG

6481 AGTTAATGCA GAAGCATATA CATTCTCCTG AAGTGGCTGA AAGTGGCTGT AAAATGCTAA
6541 ATCATCTTTT TGAAGGAAGC AACACTTCCC TGGATATAAT GGCAGCAGTG GTCCCCAAAA
6601 TACTAACAGT TATGAAACGT CATGAGACAT CATTACCAGT GCAGCTGGAG GCGCTTCGAG
6661 CTATTTTACA TTTTATAGTG CCTGGCATGC CAGAAGAATC CAGGGAGGAT ACAGAATTTC
6721 ATCATAAGCT AAATATGGTT AAAAAACAGT GTTTCAAGAA TGATATTCAC AAACTGGTCC
6781 TAGCAGCTTT GAACAGGTTC ATTGGAAATC CTGGGATTCA GAAATGTGGA TTAAAAGTAA
6841 TTTCTTCTAT TGTACATTTT CCTGATGCAT TAGAGATGTT ATCCCTGGAA GGTGCTATGG
6901 ATTCAGTGCT TCACACACTG CAGATGTATC CAGATGACCA AGAAATTCAG TGTCTGGGTT
6961 TAAGTCTTAT AGGATACTTG ATTACAAAGA AGAATGTGTT CATAGGAACT GGACATCTGC
7021 TGGCAAAAAT TCTGGTTTCC AGCTTATACC GATTTAAGGA TGTTGCTGAA ATACAGACTA
7081 AAGGATTTCA GACAATCTTA GCAATCCTCA AATTGTCAGC ATCTTTTTCT AAGCTGCTGG
7141 TGCATCATTC ATTTGACTTA GTAATATTCC ATCAAATGTC TTCCAATATC ATGGAACAAA
7201 AGGATCAACA GTTTCTAAAC CTCTGTTGCA AGTGTTTTGC AAAAGTAGCT ATGGATGATT
7261 ACTTAAAAAA TGTGATGCTA GAGAGAGCGT GTGATCAGAA TAACAGCATC ATGGTTGAAT
7321 GCTTGCTTCT ATTGGGAGCA GATGCCAATC AAGCAAAGGA GGGATCTTCT TTAATTTGTC
7381 AGGTATGTGA GAAAGAGAGC AGTCCCAAAT TGGTGGAACT CTTACTGAAT AGTGGATCTC
7441 GTGAACAAGA TGTACGAAAA GCGTTGACGA TAAGCATTGG GAAAGGTGAC AGCCAGATCA
7501 TCAGCTTGCT CTTAAGGAGG CTGGCCCTGG ATGTGGCCAA CAATAGCATT TGCCTTGGAG
7561 GATTTTGTAT AGGAAAAGTT GAACCTTCTT GGCTTGGTCC TTTATTTCCA GATAAGACTT
7621 CTAATTTAAG GAAACAAACA AATATAGCAT CTACACTAGC AAGAATGGTG ATCAGATATC
7681 AGATGAAAAG TGCTGTGGAA GAAGGAACAG CCTCAGGCAG CGATGGAAAT TTTTCTGAAG
7741 ATGTGCTGTC TAAATTTGAT GAATGGACCT TTATTCCTGA CTCTTCTATG GACAGTGTGT
7801 TTGCTCAAAG TGATGACCTG GATAGTGAAG GAAGTGAAGG CTCATTTCTT GTGAAAAAGA
7861 AATCTAATTC AATTAGTGTA GGAGAATTTT ACCGAGATGC CGTATTACAG CGTTGCTCAC
7921 CAAATTTGCA AAGACATTCC AATTCCTTGG GGCCCATTTT TGATCATGAA GATTTACTGA
7981 AGCGAAAAAG AAAAATATTA TCTTCAGATG ATTCACTCAG GTCATCAAAA CTTCAATCCC
8041 ATATGAGGCA TTCAGACAGC ATTTCTTCTC TGGCTTCTGA GAGAGAATAT ATTACATCAC
8101 TAGACCTTTC AGCAAATGAA CTAAGAGATA TTGATGCCCT AAGCCAGAAA TGCTGTATAA
8161 GTGTTCATTT GGAGCATCTT GAAAAGCTGG AGCTTCACCA GAATGCACTC ACGAGCTTTC
8221 CACAACAGCT ATGTGAAACT CTGAAGAGTT TGACACATTT GGACTTGCAC AGTAATAAAT
8281 TTACATCATT TCCTTCTTAT TTGTTGAAAA TGAGTTGTAT TGCTAATCTT GATGTCTCTC
8341 GAAATGACAT TGGACCCTCA GTGGTTTTAG ATCCTACAGT GAAATGTCCA ACTCTGAAAC
8401 AGTTTAACCT GTCATATAAC CAGCTGTCTT TTGTACCTGA GAACCTCACT GATGTGGTAG
8461 AGAAACTGGA GCAGCTCATT TTAGAAGGAA ATAAAATATC AGGGATATGC TCCCCCTTGA
8521 GACTGAAGGA ACTGAAGATT TTAAACCTTA GTAAGAACCA CATTTCATCC CTATCAGAGA
8581 ACTTTCTTGA GGCTTGTCCT AAAGTGGAGA GTTTCAGTGC CAGAATGAAT TTTCTTGCTG
8641 CTATGCCTTT CTTGCCTCCT TCTATGACAA TCCTAAAATT ATCTCAGAAC AAATTTTCCT
8701 GTATTCCAGA AGCAATTTTA AATCTTCCAC ACTTGCGGTC TTTAGATATG AGCAGCAATG
8761 ATATTCAGTA CCTACCAGGT CCCGCACACT GGAAATCTTT GAACTTAAGG GAACTCTTAT
8821 TTAGCCATAA TCAGATCAGC ATCTTGGACT TGAGTGAAAA AGCATATTTA TGGTCTAGAG
8881 TAGAGAAACT GCATCTTTCT CACAATAAAC TGAAAGAGAT TCCTCCTGAG ATTGGCTGTC
8941 TTGAAAATCT GACATCTCTG GATGTCAGTT ACAACTTGGA ACTAAGATCC TTTCCCAATG
9001 AAATGGGGAA ATTAAGCAAA ATATGGGATC TTCCTTTGGA TGAACTGCAT CTTAACTTTG
9061 ATTTTAAACA TATAGGATGT AAAGCCAAAG ACATCATAAG GTTTCTTCAA CAGCGATTAA
9121 AAAAGGCTGT GCCTTATAAC CGAATGAAAC TTATGATTGT GGGAAATACT GGGAGTGGTA
9181 AAACCACCTT ATTGCAGCAA TTAATGAAAA CCAAGAAATC AGATCTTGGA ATGCAAAGTG
9241 CCACAGTTGG CATAGATGTG AAAGACTGGC CTATCCAAAT AAGAGACAAA AGAAAGAGAG

9301 ATCTCGTCCT AAATGTGTGG GATTTTGCAG GTCGTGAGGA ATTCTATAGT ACTCATCCCC
9361 ATTTTATGAC GCAGCGAGCA TTGTACCTTG CTGTCTATGA CCTCAGCAAG GGACAGGCTG
9421 AAGTTGATGC CATGAAGCCT TGGCTCTTCA ATATAAAGGC TCGCGCTTCT TCTTCCCCTG
9481 TGATTCTCGT TGGCACACAT TTGGATGTTT CTGATGAGAA GCAACGCAAA GCCTGCATGA
9541 GTAAAATCAC CAAGGAACTC CTGAATAAGC GAGGGTTCCC TGCCATACGA GATTACCACT
9601 TTGTGAATGC CACCGAGGAA TCTGATGCTT TGGCAAAACT TCGGAAAACC ATCATAAACG
9661 AGAGCCTTAA TTTCAAGATC CGAGATCAGC TTGTTGTTGG ACAGCTGATT CCAGACTGCT
9721 ATGTAGAACT TGAAAAAATC ATTTTATCGG AGCGTAAAAA TGTGCCAATT GAATTTCCCG
9781 TAATTGACCG GAAACGATTA TTACAACTAG TGAGAGAAAA TCAGCTGCAG TTAGATGAAA
9841 ATGAGCTTCC TCACGCAGTT CACTTTCTAA ATGAATCAGG AGTCCTTCTT CATTTTCAAG
9901 ACCCAGCACT GCAGTTAAGT GACTTGTACT TTGTGGAACC CAAGTGGCTT TGTAAAATCA
9961 TGGCACAGAT TTTGACAGTG AAAGTGGAAG GTTGTCCAAA ACACCCTAAG GGCATTATTT
10021 CGCCTAGAGA TGTGGAAAAA TTTCTTTCAA AAAAAAGGAA ATTTCCAAAG AACTACATGT
10081 CACAGTATTT TAAGCTCCTA GAAAAATTCC AGATTGCTTT GCCAATAGGA GAAGAATATT
10141 TGCTGGTTCC AAGCAGTTTG TCTGACCACA GGCCTGTGAT AGAGCTTCCC CATTGTGAGA
10201 ACTCTGAAAT TATCATCCGA CTATATGAAA TGCCTTATTT TCCAATGGGA TTTTGGTCAA
10261 GATTAATCAA TCGATTACTT GAGATTTCAC CTTACATGCT TTCAGGGAGA GAACGAGCAC
10321 TTCGCCCAAA CAGAATGTAT TGGCGACAAG GCATTTACTT AAATTGGTCT CCTGAAGCTT
10381 ATTGTCTGGT AGGATCTGAA GTCTTAGACA ATCATCCAGA GAGTTTCTTA AAAATTACAG
10441 TTCCTTCTTG TAGAAAAGGC TGTATTCTTT TGGGCCAAGT TGTGGACCAC ATTGATTCTC
10501 TCATGGAAGA ATGGTTTCCT GGGTTGCTGG AGATTGATAT TTGTGGTGAA GGAGAAACTC
10561 TGTTGAAGAA ATGGGCATTA TATAGTTTTA ATGATGGTGA AGAACATCAA AAAATCTTAC
10621 TTGATGACTT GATGAAGAAA GCAGAGGAAG GAGATCTCTT AGTAAATCCA GATCAACCAA
10681 GGCTCACCAT TCCAATATCT CAGATTGCCC CTGACTTGAT TTTGGCTGAC CTGCCTAGAA
10741 ATATTATGTT GAATAATGAT GAGTTGGAAT TTGAACAAGC TCCAGAGTTT CTCCTAGGTG
10801 ATGGCAGTTT TGGATCAGTT TACCGAGCAG CCTATGAAGG AGAAGAAGTG GCTGTGAAGA
10861 TTTTTAATAA ACATACATCA CTCAGGCTGT TAAGACAAGA GCTTGTGGTG CTTTGCCACC
10921 TCCACCACCC CAGTTTGATA TCTTTGCTGG CAGCTGGGAT TCGTCCCCGG ATGTTGGTGA
10981 TGGAGTTAGC CTCCAAGGGT TCCTTGGATC GCCTGCTTCA GCAGGACAAA GCCAGCCTCA
11041 CTAGAACCCT ACAGCACAGG ATTGCACTCC ACGTAGCTGA TGGTTTGAGA TACCTCCACT
11101 CAGCCATGAT TATATACCGA GACCTGAAAC CCCACAATGT GCTGCTTTTC ACACTGTATC
11161 CCAATGCTGC CATCATTGCA AAGATTGCTG ACTACGGCAT TGCTCAGTAC TGCTGTAGAA
11221 TGGGGATAAA AACATCAGAG GGCACACCAG GGTTTCGTGC ACCTGAAGTT GCCAGAGGAA
11281 ATGTCATTTA TAACCAACAG GCTGATGTTT ATTCATTTGG TTTACTACTC TATGACATTT
11341 TGACAACTGG AGGTAGAATA GTAGAGGGTT TGAAGTTTCC AAATGAGTTT GATGAATTAG
11401 AAATACAAGG AAAATTACCT GATCCAGTTA AAGAATATGG TTGTGCCCCA TGGCCTATGG
11461 TTGAGAAATT AATTAAACAG TGTTTGAAAG AAAATCCTCA AGAAAGGCCT ACTTCTGCCC
11521 AGGTCTTTGA CATTTTGAAT TCAGCTGAAT TAGTCTGTCT GACGAGACGC ATTTTATTAC
11581 CTAAAAACGT AATTGTTGAA TGCATGGTTG CTACACATCA CAACAGCAGG AATGCAAGCA
11641 TTTGGCTGGG CTGTGGGCAC ACCGACAGAG GACAGCTCTC ATTTCTTGAC TTAAATACTG
11701 AAGGATACAC TTCTGAGGAA GTTGCTGATA GTAGAATATT GTGCTTAGCC TTGGTGCATC
11761 TTCCTGTTGA AAAGGAAAGC TGGATTGTGT CTGGGACACA GTCTGGTACT CTCCTGGTCA
11821 TCAATACCGA AGATGGGAAA AAGAGACATA CCCTAGAAAA GATGACTGAT TCTGTCACTT
11881 GTTTGTATTG CAATTCCTTT TCCAAGCAAA GCAAACAAAA AAATTTTCTT TTGGTTGGAA
11941 CCGCTGATGG CAAGTTAGCA ATTTTTGAAG ATAAGACTGT TAAGCTTAAA GGAGCTGCTC
12001 CTTTGAAGAT ACTAAATATA GGAAATGTCA GTACTCCATT GATGTGTTTG AGTGAATCCA
12061 CAAATTCAAC GGAAAGAAAT GTAATGTGGG GAGGATGTGG CACAAAGATT TTCTCCTTTT

12121 CTAATGATTT CACCATTCAG AAACTCATTG AGACAAGAAC AAGCCAACTG TTTTCTTATG
12181 CAGCTTTCAG TGATTCCAAC ATCATAACAG TGGTGGTAGA CACTGCTCTC TATATTGCTA
12241 AGCAAAATAG CCCTGTTGTG GAAGTGTGGG ATAAGAAAAC TGAAAAACTC TGTGGACTAA
12301 TAGACTGCGT GCACTTTTTA AGGGAGGTAA TGGTAAAAGA AAACAAGGAA TCAAAACACA
12361 AAATGTCTTA TTCTGGGAGA GTGAAAACCC TCTGCCTTCA GAAGAACACT GCTCTTTGGA
12421 TAGGAACTGG AGGAGGCCAT ATTTTACTCC TGGATCTTTC AACTCGTCGA CTTATACGTG
12481 TAATTTACAA CTTTTGTAAT TCGGTCAGAG TCATGATGAC AGCACAGCTA GGAAGCCTTA
12541 AAAATGTCAT GCTGGTATTG GGCTACAACC GGAAAAATAC TGAAGGTACA CAAAAGCAGA
12601 AAGAGATACA ATCTTGCTTG ACCGTTTGGG ACATCAATCT TCCACATGAA GTGCAAAATT
12661 TAGAAAAACA CATTGAAGTG AGAAAAGAAT TAGCTGAAAA AATGAGACGA ACATCTGTTG
12721 AGTAATGATA GGTCGACCGC GGATCTAGAG ATCTGATAAT TCCGATCATA TTCAATAACC
12781 CTTAATATAA CTTCGTATAA TGTATGCTAT ACGAAGTTAT TAGGTCTGAA GAGGAGTTTA
12841 CGTCCAGCCA AGCTAGCTTC TCTGCAGGAT TCGAGGGCCC CTGCAGGTCA ATTCTACCGG
12901 GTAGGGGAGG CGCTTTTCCC AAGGCAGTCT GGAGCATGCG CTTTAGCAGC CCCGCTGGCA
12961 CTTGGCGCTA CACAAGTGGC CTCTGGCCTC GCACACATTC CACATCCACC GGTAGCGCCA
13021 ACCGGCTCCG TTCTTTGGTG GCCCCTTCGC GCCACCTTCT ACTCCTCCCC TAGTCAGGAA
13081 GTTCCCCCCC GCCCCGCAGC TCGCGTCGTG CAGGACGTGA CAAATGGAAG TAGCACGTCT
13141 CACTAGTCTC GTGCAGATGG ACAGCACCGC TGAGCAATGG AAGCGGGTAG GCCTTTGGGG
13201 CAGCGGCCAA TAGCAGCTTT GCTCCTTCGC TTTCTGGGCT CAGAGGCTGG GAAGGGGTGG
13261 GTCCGGGGGC GGGCTCAGGG GCGGGCTCAG GGGCGGGGCG GGCGCGAAGG TCCTCCCGAG
13321 GCCCGGCATT CTCGCACGCT TCAAAAGCGC ACGTCTGCCG CGCTGTTCTC CTCTTCCTCA
13381 TCTCCGGGCC TTTCGACCTG CAGCCAATAT GGGATCGGCC ATTGAACAAG ATGGATTGCA
13441 CGCAGGTTCT CCGGCCGCTT GGGTGGAGAG GCTATTCGGC TATGACTGGG CACAACAGAC
13501 AATCGGCTGC TCTGATGCCG CCGTGTTCCG GCTGTCAGCG CAGGGGCGCC CGGTTCTTTT
13561 TGTCAAGACC GACCTGTCCG GTGCCCTGAA TGAACTGCAG GACGAGGCAG CGCGGCTATC
13621 GTGGCTGGCC ACGACGGGCG TTCCTTGCGC AGCTGTGCTC GACGTTGTCA CTGAAGCGGG
13681 AAGGGACTGG CTGCTATTGG GCGAAGTGCC GGGGCAGGAT CTCCTGTCAT CTCACCTTGC
13741 TCCTGCCGAG AAAGTATCCA TCATGGCTGA TGCAATGCGG CGGCTGCATA CGCTTGATCC
13801 GGCTACCTGC CCATTCGACC ACCAAGCGAA ACATCGCATC GAGCGAGCAC GTACTCGGAT
13861 GGAAGCCGGT CTTGTCGATC AGGATGATCT GGACGAAGAG CATCAGGGGC TCGCGCCAGC
13921 CGAACTGTTC GCCAGGCTCA AGGCGCGCAT GCCCGACGGC GAGGATCTCG TCGTGACCCA
13981 TGGCGATGCC TGCTTGCCGA ATATCATGGT GGAAAATGGC CGCTTTTCTG GATTCATCGA
14041 CTGTGGCCGG CTGGGTGTGG CGGACCGCTA TCAGGACATA GCGTTGGCTA CCCGTGATAT
14101 TGCTGAAGAG CTTGGCGGCG AATGGGCTGA CCGCTTCCTC GTGCTTTACG GTATCGCCGC
14161 TCCCGATTCG CAGCGCATCG CCTTCTATCG CCTTCTTGAC GAGTTCTTCT GAGGGGATCG
14221 ATCCGTCCTG TAAGTCTGCA GAAATTGATG ATCTATTAAA CAATAAAGAT GTCCACTAAA
14281 ATGGAAGTTT TTCCTGTCAT ACTTTGTTAA GAAGGGTGAG AACAGAGTAC CTACATTTTG
14341 AATGGAAGGA TTGGAGCTAC GGGGGTGGGG GTGGGGTGGG ATTAGATAAA TGCCTGCTCT
14401 TTACTGAAGG CTCTTTACTA TTGCTTTATG ATAATGTTTC ATAGTTGGAT ATCATAATTT
14461 AAACAAGCAA AACCAAATTA AGGGCCAGCT CATTCCTCCC ACTCATGATC TATAGATCTA
14521 TAGATCTCTC GTGGGATCAT TGTTTTTCTC TTGATTCCCA CTTTGTGGTT CTAAGTACTG
14581 TGGTTTCCAA ATGTGTCAGT TTCATAGCCT GAAGAACGAG ATCAGCAGCC TCTGTTCCAC
14641 ATACACTTCA TTCTCAGTAT TGTTTTGCCA AGTTCTAATT CCATCAGAAG CTGACTCTAG
14701 ATCTGGATCG ATCCGGAACC CTTAATATAA CTTCGTATAA TGTATGCTAT ACGAAGTTAT
14761 TAGGTCCCTC GAAGAGGTTC ACTAGTACTG GCCAATCGGC GCGCCGGAGG AGGCTCTGAA
14821 GAAGTTGATA GTCAGGCTGA ATAATGTCCA GGAAGGCAAG CAGATCGAGA CGTTGCTTCA
14881 GCTCCTGGAG GACATGCTGG TGTTCACCTA CTCGGACCGC GGTAATGCCT GCAGCCCCTC

14941 CCTCCTCTGC AAACTTTATC CTGCACTTGA GTCTTAATCT TGGGCACCCC ACTCCTTTCC
15001 TTCAGGGAGG AAGGAGCTGA GATCCCTGGA AGATTGAAAG GATGCCAGGA TGCCGTGGTT
15061 TGTGACTTTG TTTTCATCCC CCATTTGCCC CCTTCTTTTC CAGCCTCCAA GTTATTTGAA
15121 GATAAAAATT TCCACGTGCC TCTGTTGATT GTCCTGGACT CCTACATGAG AGTTGCCAGT
15181 GTACAGCAGG TAAGCGGGCC TTCCCATCTG TTCCTGAAGA CTGTTCTGTG ATAGGCCTTA
15241 TGTCCCGGGG TCTTGAGATT TGCTTTAAGA GGGGAAATCT TGTCAAATGT TGGCAGAGGA
15301 GAAGCACCTT TGACATTATC TGGGTGGTGG TAGCAGCAAA ATGAAAACAG GGCTCTTATT
15361 GGCAAGGTGA GTCCGTTAAC CTTTCAAACT ATTCTAAGAA TGCTCCTTAA TATACAGCGA
15421 GTTTCTGGTA GAGCAGTGCA GTGAATATTT CCAGCAAGGG AGGGTAATTA AAAAAACAGT
15481 AATAAAACAG TCAAGTCTAG GGTCAGTTTG CTTTTATACT TTTAAATTAG AAGACACATT
15541 TCAAGGATTC TTCCTGCAGT GTATATCACC CAGTATCTAA AGAAAGTGAT GTTTCCCGAA
15601 TGCCCCCTCG CCTGCCGTGT TTACATTTCT ACTTTGCGGC TGTTATCATA CAAAGTGGAT
15661 ACAAAAGTAG AACTTCTGTC TGGATCTGTG TCACAGGAAG TCCAGAAGTA GGCTATGTAA
15721 TTAACTGTGA TTTTAAAGCA GACTCGGATA AGTAAGAATG GCTTTCAGTC AGGACAGAAT
15781 TACTTCTGAA GGTGCTAACT TCCTTTAGAG GTTTGCGATC TGGGGAACTG AAATCAGTTT
15841 TGTCATCCAA ATACTGAGAG TTATTTGTCG GGGAACTCTG GAGGCACGGG AAGAATAGAA
15901 TACTCCTTCA TAGTCATTCT AGAGACTAAA CTTTTCTTAG TCTCCAAGTT CTCTCTCTCT
15961 CTCTCTCTCT CTCTCTCTCT CTCTCTCTCT CTCTCTCTCT CCTCCTCCTC CTCCTCCTCC
16021 TCTCCTGCCT CCTCCTCTTT CTCCCTCTGT TCCCACCCTG TCAAGTTCTC ACAGTATAAC
16081 CCAGACTACT TAGAACTTGC CATTTTCCTG CCTCTGGCTC CCCAGTGCTA TAATTATAGG
16141 TATATGTCAC CATACCTGGC TCTAATTTTC TTTTAGAAAA GCATACTTTT ATCTGCAGTA
16201 AATATTTTTA TTTTTCCAAT TGTGAAACTT TCCTATCTGA CTAGTCATTT TCAGGTTGTT
16261 ATCAGACAAA AATCAAGAGG AAGATGGAGG TATGATAAAG ATACTGTACT TTTTAGCACC
16321 AAGAGTCTGA ATTTTACCCA CGGAATATGA ATCATTTTGA AGGTGCTTTC TGTTCTCCAC
16381 CTACATCACC CTTCTTGCTT TATATTCAAA AGTGTCTAAT TCTGGCTGCC TACACTGCCA
16441 AGACCTAGAC TTGGTAGTCT CACGCCTGCT TGGTACCACT GGGAAAAACT GGACCCTGAT
16501 TTTCAATCAA CTGAACACAG AAGGGAGTGG TAGACCTGAG AGCCCAGGGG AATCTGGAAT
16561 ATAGCCATTG CTTACCTCTT TCTCCTTTCA TCTCTTCCTT TTGTGTCTGT CTGTCTTTGC
16621 TCTGTCTCTG TCTCTCCTCC TTCTTTCTCT GTGCTGACAA GATCTGTGAC TTTAAATCTG
16681 CTAATGGAAT TACTTTGATT ACTGATCTAT ATCCCCAAAC ACTGTAATTT TCTCTTCCCT
16741 GTCTCCCCCC TAACTTTCCT CTTTCATTCC CCTTATCCAT TTCTACTCCA CTCCCTTTTT
16801 CTTCTTCATT CTCTCTTCCT CCTTCTCCTT CTCCTCCTCC TCCATTTTCT CCTCCTTCTT
16861 TCCACCCTCC TCCTCCTCCA CTGCATTTTT GGTGCTGGGA ATTAAACACA GGGATTTGCA
16921 TCTACCGGAC AAGCTCTCTA CCAGTAAGCT ACCCAGTCAA CCAAGACATT CTAGTTCTTG
16981 ATTTTAGAAA ATGCCCAAGT AACTGCAGTG GTTAAGATAT TGTTACCAGC TTGTGGAGTG
17041 GGCAGCTGCA AAGTCTATTG GAAAGGAACA GCCTCTGGTT TGATAATTTA GCAGAATAAA
17101 GTCAGTACTT TCAGTGTTTT ATATGTGTTT ATTTGCAAGG GCTTTATGCA TATTATGTTA
17161 ATTGTTTTTT CTCTTTGCGG TTCTTTATTA GATATTTTCT TTATATACAT TTCAAATGCT
17221 ACCCCGAAAG TTCCCTATAC CCTCCCTCAA CCCTGCTCCC CTACCCACCC ACTCCTTCTT
17281 CTTGGCCCTG GCATTCCCCT GTACTGGGGC ATATAAAGTT TGCAATACCA AGGGGCCTCT
17341 CTTCCCAGTG ATGGGCGACT AGGCCATCTT CTGCTACATA TGCAGCTAGA GACAGTAGTT
17401 CTGGGGGTAC TGGTTAGTTC ATATTGTTGT TTCACCTATA GGGTTGCAAG ACCCCTTCAG
17461 CTCCTTGGGT GCTTTCTCTA GCTTCTCCAT TGGGGGCCCT GTGATCCATC TTATAGATGA
17521 TTGTCTCATA TGTTAATTGG TTCTTACTGA CATTTGATTG CACTTGAAGA AAGTTGACCA
17581 GAGTCTTTCC TTCATATTGA TTTAGTTAAA ATGAGTAGTC CCTGTGGATA CTTGTAATAA
17641 AGCTAGGGCT CAAATATGTT CGAGTCTTCA AAATCTCCAT CATTTAGTTC TGGACTTACT
17701 AAGATTTTTG TGTGAGCAAG CAAAGTCCAA TGGTTTGGCA CAAATACTAT GAGCAAATAC

17761 AATGGCTATA TTGCCGTTGG TCTTATCAAA GGGTTATAGA AAGGATTCTT TCTCTACTGA
17821 GATTTTTAGC ATCTCTAATT GAAAGTAATA TCTTCTTTTA AATCTCTTCA CATAATTGGT
17881 GAAAGTAGTC TGAGCACCCT TCCATAGTTC TTGCAATCCA ATTAGCTAGA AGCACCAAAT
17941 GAGAATGCCT TGGTGAAGGC AAGGAGTTCC TCGTCAGACA TGTATGTTAA GGTTTCTTGT
18001 GAGCATCATC CAACACGGCA TTTGGGATTC TGGACTTTTA CAATTTGCTT TATCTGTGCC
18061 CTTTTACAGT TGCAGATCTC CTAGGGCTGC TCTCAAGCTT GCCCTGTGCA TGTCTCCTCC
18121 TGTTAGTCAT TCTATTTTGC TACTCAATTT GACTTCTCTT TTTCTACGTC ATTCCAACCT
18181 TTGAAGGAAG AGAGCAAAAT AAATTAAGTG ACTTAAATGT TCTCCAACCC TCTGATTTTT
18241 TTTCACATCA TTTATGAATG GGATGCTTGA GTTTTATATC GTCCTCGTAG TAGAGGATAA
18301 CAGCCCTGTC GCGACGCGTG CTAGCCCGGG CGCCTAGTTA TTAATAGTAA TCAATTACGG
18361 GGTCATTAGT TCATAGCCCA TATATGGAGT TCCGCGTTAC ATAACTTACG GTAAATGGCC
18421 CGCCTGGCTG ACCGCCCAAC GACCCCCGCC CATTGACGTC AATAATGACG TATGTTCCCA
18481 TAGTAACGCC AATAGGGACT TTCCATTGAC GTCAATGGGT GGAGTATTTA CGGTAAACTG
18541 CCCACTTGGC AGTACATCAA GTGTATCATA TGCCAAGTAC GCCCCCTATT GACGTCAATG
18601 ACGGTAAATG GCCCGCCTGG CATTATGCCC AGTACATGAC CTTATGGGAC TTTCCTACTT
18661 GGCAGTACAT CTACGTATTA GTCATCGCTA TTACCATGGT CGAGGTGAGC CCCACGTTCT
18721 GCTTCACTCT CCCCATCTCC CCCCCCTCCC CACCCCCAAT TTTGTATTTA TTTATTTTTT
18781 AATTATTTTG TGCAGCGATG GGGGCGGGGG GGGGGGGGGG GCGCGCGCCA GGCGGGGCGG
18841 GGCGGGGCGA GGGGCGGGGC GGGGCGAGGC GGAGAGGTGC GGCGGCAGCC AATCAGAGCG
18901 GCGCGCTCCG AAAGTTTCCT TTTATGGCGA GGCGGCGGCG GCGGCGGCCC TATAAAAAGC
18961 GAAGCGCGCG GCGGGCGGCT GCGACCTGCA GGTCCTCGCC ATGGACCCTG ATGATGTTGT
19021 TGATTCTTCT AAATCTTTTG TGATGGAAAA CTTTTCTTCG TACCACGGGA CTAAACCTGG
19081 TTATGTAGAT TCCATTCAAA AAGGTATACA AAAGCCAAAA TCTGGTACAC AAGGAAATTA
19141 TGACGATGAT TGGAAAGGGT TTTATAGTAC CGACAATAAA TACGACGCTG CGGGATACTC
19201 TGTAGATAAT GAAAACCCGC TCTCTGGAAA AGCTGGAGGC GTGGTCAAAG TGACGTATCC
19261 AGGACTGACG AAGGTTCTCG CACTAAAAGT GGATAATGCC GAAACTATTA AGAAAGAGTT
19321 AGGTTTAAGT CTCACTGAAC CGTTGATGGA GCAAGTCGGA ACGGAAGAGT TTATCAAAAG
19381 GTTCGGTGAT GGTGCTTCGC GTGTAGTGCT CAGCCTTCCC TTCGCTGAGG GGAGTTCTAG
19441 CGTTGAATAT ATTAATAACT GGGAACAGGC GAAAGCGTTA AGCGTAGAAC TTGAGATTAA
19501 TTTTGAAACC CGTGGAAAAC GTGGCCAAGA TGCGATGTAT GAGTATATGG CTCAAGCCTG
19561 TGCAGGAAAT CGTGTCAGGC GATCTCTTTG TGAAGGAAAC CTTACTTCTG TGGTGTGACA
19621 TAATTGGACA AACTACCTAC AGAGATTTAA AGCTCTAAGG TAAATATAAA ATTTTTAAGT
19681 GTATAATGTG TTAAACTACT GATTCCTAAT TGTTTGTGTA TTTTAGATTC CAACCTATGG
19741 AACTGATGAA TGGGAGCAGT GGTGGAATGC AGATCCACTA GGATCTAACT TGTTTATTGC
19801 AGCTTATAAT GGTTACAAAT AAAGCAATAG CATCACAAAT TTCACAAATA AAGCATTTTT
19861 TTCACTGCAT TCTAGTTGTG GTTTGTCCAA ACTCATCAAT GTATCTTATC ATGTCTGGAT
19921 CGTAGTTCTA GAGCGGACCG AGGGGGCCCG TACTACGCCT TAAGTGAGTC GTATTACGGA
19981 CTGGCCGTCG TTTTACAACG TCGTGACTGG GAAAACCCTG GCGTTACCCA ACTTAATCGC
20041 CTTGCAGCAC ATCCCCCTTT CGCCAGCTGG CGTAATAGCG AAGAGGCCCG CACCGATCGC
20101 CCTTCCCAAC AGTTGCGCAG CCTGAATGGC GAATGGCGCT TCGCTTGGTA ATAAAGCCCG
20161 CTTCGGCGGG CTTTTTTTTG TTAACTACGT CAGGTGGCAC TTTTCGGGGA AATGTGCGCG
20221 GAACCCCTAT TTGTTTATTT TTCTAAATAC ATTCAAATAT GTATCCGCTC ATGAGACAAT
20281 AACCCTGATA AATGCTTCAA TAATATTGAA AAAGGAAGAG TATGAGTATT CAACATTTCC
20341 GTGTCGCCCT TATTCCCTTT TTTGCGGCAT TTTGCCTTCC TGTTTTTGCT CACCCAGAAA
20401 CGCTGGTGAA AGTAAAAGAT GCTGAAGATC AGTTGGGTGC ACGAGTGGGT TACATCGAAC
20461 TGGATCTCAA CAGCGGTAAG ATCCTTGAGA GTTTTCGCCC CGAAGAACGT TCTCCAATGA
20521 TGAGCACTTT TAAAGTTCTG CTATGTGGCG CGGTATTATC CCGTGTTGAC GCCGGGCAAG

20581 AGCAACTCGG TCGCCGCATA CACTATTCTC AGAATGACTT GGTTGAGTAC TCACCAGTCA
20641 CAGAAAAGCA TCTTACGGAT GGCATGACAG TAAGAGAATT ATGCAGTGCT GCCATAACCA
20701 TGAGTGATAA CACTGCGGCC AACTTACTTC TGACAACGAT CGGAGGACCG AAGGAGCTAA
20761 CCGCTTTTTT GCACAACATG GGGGATCATG TAACTCGCCT TGATCGTTGG GAACCGGAGC
20821 TGAATGAAGC CATACCAAAC GACGAGCGTG ACACCACGAT GCCTGTAGCA ATGGCAACAA
20881 CGTTGCGCAA ACTATTAACT GGCGAACTAC TTACTCTAGC TTCCCGGCAA CAATTAATAG
20941 ACTGGATGGA GGCGGATAAA GTTGCAGGAC CACTTCTGCG CTCGGCCCTT CCGGCTGGCT
21001 GGTTTATTGC TGATAAATCT GGAGCCGGTG AGCGTGGGTC TCGCGGTATC ATTGCAGCAC
21061 TGGGGCCAGA TGGTAAGCCC TCCCGTATCG TAGTTATCTA CACGACGGGG AGTCAGGCAA
21121 CTATGGATGA ACGAAATAGA CAGATCGCTG AGATAGGTGC CTCACTGATT AAGCATTGGT
21181 AACTGTCAGA CCAAGTTTAC TCATATATAC TTTAGATTGA TTTACCCCGG TTGATAATCA
21241 GAAAAGCCCC AAAAACAGGA AGATTGTATA AGCAAATATT TAAATTGTAA ACGTTAATAT
21301 TTTGTTAAAA TTCGCGTTAA ATTTTTGTTA AATCAGCTCA TTTTTTAACC AATAGGCCGA
21361 AATCGGCAAA ATCCCTTATA AATCAAAAGA ATAGCCCGAG ATAGGGTTGA GTGTTGTTCC
21421 AGTTTGGAAC AAGAGTCCAC TATTAAAGAA CGTGGACTCC AACGTCAAAG GGCGAAAAAC
21481 CGTCTATCAG GGCGATGGCC CACTACGTGA ACCATCACCC AAATCAAGTT TTTTGGGGTC
21541 GAGGTGCCGT AAAGCACTAA ATCGGAACCC TAAAGGGAGC CCCCGATTTA GAGCTTGACG
21601 GGGAAAGCGA ACGTGGCGAG AAAGGAAGGG AAGAAAGCGA AAGGAGCGGG CGCTAGGGCG
21661 CTGGCAAGTG TAGCGGTCAC GCTGCGCGTA ACCACCACAC CCGCCGCGCT TAATGCGCCG
21721 CTACAGGGCG CGTAAAAGGA TCTAGGTGAA GATCCTTTTT GATAATCTCA TGACCAAAAT
21781 CCCTTAACGT GAGTTTTCGT TCCACTGAGC GTCAGACCCC GTAGAAAAGA TCAAAGGATC
21841 TTCTTGAGAT CCTTTTTTTC TGCGCGTAAT CTGCTGCTTG CAAACAAAAA AACCACCGCT
21901 ACCAGCGGTG GTTTGTTTGC CGGATCAAGA GCTACCAACT CTTTTTCCGA AGGTAACTGG
21961 CTTCAGCAGA GCGCAGATAC CAAATACTGT TCTTCTAGTG TAGCCGTAGT TAGGCCACCA
22021 CTTCAAGAAC TCTGTAGCAC CGCCTACATA CCTCGCTCTG CTAATCCTGT TACCAGTGGC
22081 TGCTGCCAGT GGCGATAAGT CGTGTCTTAC CGGGTTGGAC TCAAGACGAT AGTTACCGGA
22141 TAAGGCGCAG CGGTCGGGCT GAACGGGGGG TTCGTGCACA CAGCCCAGCT TGGAGCGAAC
22201 GACCTACACC GAACTGAGAT ACCTACAGCG TGAGCTATGA GAAAGCGCCA CGCTTCCCGA
22261 AGGGAGAAAG GCGGACAGGT ATCCGGTAAG CGGCAGGGTC GGAACAGGAG AGCGCACGAG
22321 GGAGCTTCCA GGGGGAAACG CCTGGTATCT TTATAGTCCT GTCGGGTTTC GCCACCTCTG
22381 ACTTGAGCGT CGATTTTTGT GATGCTCGTC AGGGGGGCGG AGCCTATGGA AAAACGCCAG
22441 CAACGCGGCC TTTTTACGGT TCCTGGCCTT TTGCTGGCCT TTTGCTCACA TGTAATGTGA
22501 GTTAGCTCAC TCATTAGGCA CCCCAGGCTT TACACTTTAT GCTTCCGGCT CGTATGTTGT
22561 GTGGAATTGT GAGCGGATAA CAATTTCACA CAGGAAACAG CTATGACCAT GATTACGCCA
22621 AGCTACGTAA TACGACTCAC TAG
